# High-throughput proteomics and *in vitro* functional characterization of the 26 medically most important elapids and vipers from sub-Saharan Africa

**DOI:** 10.1093/gigascience/giac121

**Published:** 2022-12-13

**Authors:** Giang Thi Tuyet Nguyen, Carol O'Brien, Yessica Wouters, Lorenzo Seneci, Alex Gallissà-Calzado, Isabel Campos-Pinto, Shirin Ahmadi, Andreas H Laustsen, Anne Ljungars

**Affiliations:** Department of Biotechnology and Biomedicine, Technical University of Denmark, DK-2800 Kongens Lyngby, Denmark; Department of Biotechnology and Biomedicine, Technical University of Denmark, DK-2800 Kongens Lyngby, Denmark; Department of Biotechnology and Biomedicine, Technical University of Denmark, DK-2800 Kongens Lyngby, Denmark; Department of Biotechnology and Biomedicine, Technical University of Denmark, DK-2800 Kongens Lyngby, Denmark; Department of Biotechnology and Biomedicine, Technical University of Denmark, DK-2800 Kongens Lyngby, Denmark; Department of Biotechnology and Biomedicine, Technical University of Denmark, DK-2800 Kongens Lyngby, Denmark; Department of Biotechnology and Biomedicine, Technical University of Denmark, DK-2800 Kongens Lyngby, Denmark; Department of Biotechnology and Biomedicine, Technical University of Denmark, DK-2800 Kongens Lyngby, Denmark; Department of Biotechnology and Biomedicine, Technical University of Denmark, DK-2800 Kongens Lyngby, Denmark

**Keywords:** snakebite envenoming, sub-Saharan Africa, toxicovenomic, *in vitro* venom characterization, high-throughput assays, cytotoxicity, enzymatic activity of venoms

## Abstract

Venomous snakes are important parts of the ecosystem, and their behavior and evolution have been shaped by their surrounding environments over the eons. This is reflected in their venoms, which are typically highly adapted for their biological niche, including their diet and defense mechanisms for deterring predators. Sub-Saharan Africa is rich in venomous snake species, of which many are dangerous to humans due to the high toxicity of their venoms and their ability to effectively deliver large amounts of venom into their victims via their bite. In this study, the venoms of 26 of sub-Saharan Africa's medically most relevant elapid and viper species were subjected to parallelized toxicovenomics analysis. The analysis included venom proteomics and *in vitro* functional characterization of whole venom toxicities, enabling a robust comparison of venom profiles between species. The data presented here corroborate previous studies and provide biochemical details for the clinical manifestations observed in envenomings by the 26 snake species. Moreover, two new venom proteomes (*Naja anchietae* and *Echis leucogaster*) are presented here for the first time. Combined, the presented data can help shine light on snake venom evolutionary trends and possibly be used to further improve or develop novel antivenoms.

## Introduction

In the deep jungles, on the open savanna, and across deserts, snakes are omnipresent in sub-Saharan Africa, where they play an integral role in the natural ecosystems to which they have adapted over the course of evolution [1]. Some of these snake species are highly venomous, being classified by the World Health Organization as category 1 or 2 snakes of the highest medical importance [2, 3]. Thus, understanding the composition and function of their venoms is not only important for elucidating basic biology and adaptation of species, but also of medical significance. Each year, venomous snakes inflict approximately 500,000 bites in Africa [4, 5], causing major disability and disablement for many rural workers and children [6]. This challenge remains a pressing health care issue, which is further exacerbated by the socioeconomic impact that disability causes for manual laborers [7].

The medically most important snakes of sub-Saharan Africa belong mainly to the Elapidae (e.g., cobras, mambas, and rinkhals) and Viperidae families, although a few species from the Colubridae family (e.g., boomslang, *Dispholidus typus*) are also known to cause severe envenomings. Victims envenomed by elapid snakes typically display local as well as systemic clinical manifestations. Local manifestation often includes swelling, blistering, and bruising at the anatomic site of the bite, which may evolve into irreversible tissue necrosis and gangrene [5, 8]. In comparison, systemic manifestations may include muscle twitching, spasms, weakness, fatigue, sleepiness, slurred speech, or difficulties in swallowing. These can progress to flaccid paralysis and, in severe cases, fatal respiratory failure, unless mechanical ventilation is provided [5, 9].

Similarly to elapids, envenomings caused by vipers may also result in both local and systemic manifestations. The victims often immediately feel a strong irradiating pain at the site of bite and typically show hot inflammatory erythema, blisters, bruises, and spontaneous bleeding [5]. Systemic clinical manifestations can include temporary loss of vision, fainting, and systemic hemorrhage, which in severe cases can lead to cardiovascular shock [5].

Different toxin families are responsible for the clinical manifestations observed for viper and elapid envenoming. As a first example, venoms from spitting cobras are rich in cytotoxins (CTxs) from the 3-finger toxin (3FTx) family and phospholipase A_2_s (PLA_2_s) [10], which interfere with and disrupt the integrity of cellular membranes, resulting in irreversible damage and cell death. In comparison, short- and long-chain α-neurotoxins, another type of 3FTx, found in venoms such as those of the black mamba and forest cobra, block neuromuscular signaling and prevent normal muscle contractions through binding to acetylcholine receptors in neuromuscular junctions [11]. Another example of a class of toxins that interfere with neuromuscular signaling are dendrotoxins. Dendrotoxins belong to the Kunitz-type inhibitors, are exclusively found in the venoms of mambas, and block ion transport through potassium channels, resulting in involuntary muscle contractions [12, 13]. Snake venoms from most viperid species possess a high fraction of PLA_2_s (e.g., Gaboon viper, *Bitis gabonica*), snake venom metalloproteinases (SVMPs) (e.g., carpet viper, *Echis ocellatus*), and snake venom serine proteinases (SVSPs) (e.g., horned viper, *Cerastes cerastes*), which all play important roles in the toxicity of these venoms. SVMPs hydrolyze components of the cell wall of capillaries, which first reduces the mechanical integrity and then disrupts the capillary walls, resulting in both local and systemic bleeding [14, 15]. Systemic bleeding can also be caused by SVSPs that interfere with the blood coagulation cascade by decreasing the level of platelets, fibrinogen, and clotting factors [5, 16].

So far, most venomic studies on African snakes have included only one or a small handful of species [17–21], and the inclusion of functional data has been somewhat sporadic or absent. These studies have undoubtedly been important for obtaining a first snapshot of venom compositions, which have already enabled further studies within venom evolution, snake biology, and development of (recombinant) antivenom. However, the fact that these studies have been performed in multiple different laboratories using different protocols results in a limited level of data harmonization. To this end, and to elucidate a few so far undescribed venom proteomes, we describe high-throughput methods for proteomics (i.e., venomics) and *in vitro* functional characterization of snake venoms (i.e., toxicovenomics) in a parallelized manner and characterize sub-Saharan Africa's 26 medically most important snakes, comprising 18 elapids and 8 vipers (Table 1).

**Table 1:** List of the 26 venoms from the medically most relevant elapids and vipers from sub-Saharan Africa used in this study. Catalog number and origin are listed.

Family	Genus (subgenus)	Snake	Catalog No.	Origin
Elapidae	*Dendroaspis*	*D. angusticeps*	L1307	Tanzania
		*D. jamesoni*	L1308	Cameroon
		*D. polylepis*	L1309	Kenya, South Africa
		*D. viridis*	L1310	Ghana
	*Hemachatus*	*H. haemachatus*	L1311	South Africa
	*Naja (Afronaja)*	*N. ashei*	L1375	Kenya
		*N. katiensis*	L1317	Burkina Faso
		*N. mossambica*	L1376	South Africa, Tanzania
		*N. nigricincta*	L1368	South Africa
		*N. nigricollis*	L1327	Cameroon, Tanzania, West Africa
		*N. nubiae*	L1342	Egypt
		*N. pallida*	L1321	Kenya
	*Naja (Boulangerina)*	*N. melanoleuca*	L1318	Cameroon, Ghana, Uganda
	*Naja (Uraeus)*	*N. anchietae*	L1374	Namibia
		*N. annulifera*	L1314	Sub-Saharan Africa
		*N. haje*	L1315	Egypt, Mali
		*N. nivea*	L1328	South Africa
		*N. senegalensis*	L1350	Mali
Viperidae	*Bitis*	*B. arietans*	L1159	Cameroon, Kenya, Mali, Saudi Arabia, West Africa, Tanzania
		*B. gabonica*	L1104	Burundi, Tanzania
		*B. nasicornis*	L1106	West Africa, Burundi
		*B. rhinoceros*	L1105	Ghana
	*Cerastes*	*C. cerastes*	L1107	Egypt, Tunisia
	*Echis*	*E. leucogaster*	L1109	Mali
		*E. ocellatus*	L1114	Cameroon, Mali, Ghana
		*E. pyramidum*	L1110	Egypt

## Material and Methods

### Venoms and reagents

Chemicals were obtained from Sigma-Aldrich (St. Louis, MO, USA) unless otherwise stated. High-purity venoms, from a pool of specimens for the 26 snakes in Table 1, were obtained from Latoxan (Portes lés Valence, France), stored at −20°C, and reconstituted in assay buffer just before use. PLA_2_ substrate 4-nitro-3-(octanoyloxy)benzoic acid (NOB) and SVMP substrate ES010 were purchased from Enzo Life Sciences (Farmingdale, New York, USA). SVSP substrate (*p*-tosyl-Gly-Pro-Arg)_2_-R110 and 96-well plates were purchased from Thermo Fisher Scientific (Waltham, Massachusetts, USA). All substrates for enzymatic assays were dissolved in DMSO to the stock concentration of 100 mM. CellTiter-Glo 3D Cell Viability Assay kit was obtained from Promega (Madison, WI, USA).

### Reversed-phase high-performance liquid chromatography

To record the chromatograms, the venoms were separated by reversed-phase high-performance liquid chromatography (RP-HPLC) using an Agilent (Santa Clara, CA, USA) Infinity II as previously described [22]. Briefly, lyophilized venom (10 mg) was dissolved in 1 mL of water containing 0.1% trifluoroacetic acid (TFA; solution A), centrifuged at 14,000 × *g* for 10 minutes, and transferred to an HPLC vial. For each fractionation round, 100 µL of sample was injected into an RP-HPLC C18 column (250 × 4.6 mm, 5 µm particle size) and eluted at 1 mL/min by applying a gradient toward acetonitrile containing 0.1% TFA (solution B) (0–15% B for 10 minutes, 15–45% B for 60 minutes, 45–70% B for 10 minutes, and 70% B for 9 minutes).

### Proteomic characterization of whole venom by mass spectrometry

#### In-solution tryptic digestion of the venom proteins

For each of the 26 snake venoms listed in Table 1, the lyophilized whole venom was dissolved in 1× phosphate-buffered saline (PBS), and then 5 µg was vacuum dried and resuspended in 20 µL of 6 M guanidinium hydrochloride containing 10 mM TCEP, 40 mM 2-chloroacetamide, and 50 mM HEPES (pH 8.5). After adding 40 µL of digestion buffer (10% acetonitrile, 50 mM HEPES pH 8.5), samples were digested with LysC endopeptidase (1:50; w/w) for 3 hours 30 minutes at 37°C. Samples were further diluted with 140 µL of digestion buffer and mixed with trypsin (1:100; w/w). Trypsinized samples were incubated overnight at 37°C, then diluted with 200 µL of 2% TFA to quench trypsin activity. Peptides were desalted on StageTip containing Empore C18 disks, eluted in 60 µL 40% acetonitrile containing 0.1% formic acid (FA), dried in a vacuum centrifuge, and resuspended in 2% acetonitrile containing 1% TFA and iRT peptides (Biognosys, Schlieren, Switzerland).

#### Liquid chromatography/tandem mass spectrometry analysis

Mass spectrometry data were collected using a Q Exactive mass spectrometer (Thermo Fisher Scientific) coupled to a Thermo EASY-nLC 1200 liquid chromatography (LC) system (Thermo Fisher Scientific). Then, 100 ng of peptides was loaded into a 2-cm C18 trap column (Thermo Fisher Scientific, 164705) connected to a 15-cm reverse-phase analytical column (Thermo Fisher Scientific, ES900). Peptides were separated for 70 minutes with a gradient going from 10% to 60% buffer B (80% acetonitrile, 0.1% FA) over 60 minutes, until spiking to 95% buffer B for the last 10 minutes to wash the column. Full mass spectrometry (MS) spectra were collected at a resolution of 70,000, with an automatic gain control (AGC) target of 3 × 10^6^ or maximum injection time of 20 ms and a scan range of 300 to 1,750 *m/z*. The MS2 spectra were obtained at a resolution of 17,500, with an AGC target value of 1 × 10^6^ or maximum injection time of 60 ms, a normalized collision energy of 25, and an intensity threshold of 1.7 × 10^4^. Dynamic exclusion was set to 60 seconds, and ions with a charge state <2 or unassigned were excluded.

Using proteome Discoverer 2.4, peptide fragmentation spectra (MS/MS) were searched against a database consisting of all Swiss-Prot and TrEMBL protein sequences from the Serpentes suborder available in Uniprot (331,759 entries; downloaded May 2022). The search was performed using the built-in Sequest HT algorithm, which was configured to derive fully tryptic peptides using default settings. Cysteine carbamidomethyl was set as a static modification and oxidation (M), deamidation (N, Q), and acetyl on protein N-termini were set as dynamic modifications. Label-free quantitation was enabled in both processing and consensus steps, with quantitation being done using Minora Feature Detector. All results were filtered at 1% false discovery rate, and relative protein abundances were estimated by calculating the ratio of the individual protein abundances to the sum of abundances of all proteins detected within a sample.

### *In vitro* functional characterization of the whole venoms

#### PLA_2_ enzymatic activity assay

The endpoint PLA_2_ activity assay was run as described previously [23]. The snake venoms were dissolved at a concentration of 10 mg/mL in assay buffer (10 mM Tris pH 8, 100 mM NaCl, and 10 mM CaCl_2_), and a 2-fold serial dilution (10 steps) was prepared. Then, 100 µL/well of each dilution was added to a 96-well plate, together with 100 µL/well of NOB (final concentration 0.25 mM). The plates were shaken at 300 rpm for 2 minutes and then incubated at 37°C for 40 minutes. The plates were then centrifuged (3,000 × *g*, 4°C, 3 minutes) before the absorbance was recorded at 405 nm using a VICTOR Nivo plate reader (PerkinElmer, Waltham, MA, USA) at 25°C. All reactions were run in duplicates and the absorbance values were shown as averages after subtracting a blank control containing no venom. EC_50_ values (the venom concentration inducing half of the maximum absorbance at 405 nm proportional to product conversion) were determined using nonlinear fitting with sigmoidal dose–response equation of the venom dose curves analyzed by GraphPad Prism 9 software (GraphPad Software, La Jolla, CA, USA).

#### SVSP and SVMP enzymatic activity assay

To measure SVSP and SVMP activities, enzymatic assays were performed. The hydrolysis reactions were performed in 96-well plates with a final volume of 100 µL per well. The snake venoms were dissolved in PBS for SVSP or assay buffer (10 mM Tris pH 8, 100 mM NaCl, 10 mM CaCl_2_) for SVMP assays at a concentration of 10 mg/mL, and 10 dilution steps of a 2-fold serial dilution were prepared. To start the reaction, 50 µL of 2 µM SVSP substrate R110 or 10 µM SVMP substrate ES010 was mixed with 50 µL of each snake venom concentration of the serial dilution. Fluorescence data were recorded using a VICTOR Nivo plate reader at 25°C. For the SVSP assay, an excitation wavelength of 480 nm and an emission wavelength of 530 nm with 11 kinetic cycles and an interval of 90 seconds were used. For the SVMP assay, an excitation wavelength of 320 nm and an emission wavelength of 405 nm with 16 kinetic cycles and an interval of 90 seconds were used. The reactions were run in duplicate and a blank containing no venom was included.

The rate of relative fluorescence units per second (RFU/s) recorded for each venom concentration was the slope calculated from the linear fitting on its time–response curve. The rate values were then plotted against the venom concentration and a nonlinear fitting with a sigmoidal dose–response equation was used to determine the EC_50_ values (the venom concentration at which half of the maximum RFU/s rate proportional to product conversion rate was observed) using GraphPad Prism 9 software.

#### Cell viability assay

The N/TERT keratinocyte [24] cell line was cultured in Dulbecco's modified Eagle's medium (DMEM:F12, ThermoFisher Scientific, Waltham, Massachusetts, USA) supplemented with 10% (v/v) fetal bovine serum, 1% (v/v) penicillin-streptomycin, and 1× RMplus supplement [25] under standard conditions (37°C, 5% CO_2_, and 85% humidity). For the cell viability assay, cells were seeded at 4,000 cells/well in 100 µL medium and incubated overnight under standard conditions. Snake venoms were dissolved at a concentration of 10 mg/mL and then 2-fold diluted in 8 dilution steps in sterile PBS. The venom dilutions were then further diluted 1:6 in medium to the maximum concentration of 1 mg/mL and added to each well followed by a 24-hour incubation. Thereafter, the CellTiter-Glo luminescent cell viability assay [26] was used to analyze the cytotoxicity of the 26 snake venoms. The assay was performed in triplicates, with no venom as negative control (maximum viability of the cells). IC_50_ values (the venom concentration inducing 50% loss of cell viability) were determined using nonlinear fitting with the dose–response inhibition equation on the venom dose curves analyzed using GraphPad Prism 9 software.

#### Thromboelastography assay

Thromboelastography (TEG) was run for all viperid venoms (*Bitis arietans, Bitis gabonica, Bitis nasicornis, Bitis rhinoceros, Cerastes cerastes, Echis leucogaster, Echis ocellatus*, and *Echis pyramidum*) according to a protocol adapted from Seneci et al. [27] using a TEG 5000 thromboelastogram (Haemonetics, Boston, Massachusetts, USA). Solutions of 72 µL 25 mM CaCl_2_, 72 µL 0.25 mM phospholipids (Rossix, Mölndal, Sweden, catalog no. #PL052), 20 µL Tris-HCl buffer (50 mM Tris + 150 mM NaCl, pH 7.4), and 7 µL crude venom at 1 or 0.1 mg/mL in PBS (final concentration of ∼20 µg/mL and ∼2 µg/mL, respectively) were mixed in TEG disposable cups (Haemonetics). Last, 189 µL of citrated human plasma was added, and the samples were immediately run for at least 30 minutes. Negative controls were run by replacing venom with 7 µL PBS. TEG traces (3 replicates per venom concentration per species) were exported as TIFF files and processed in Adobe Photoshop 2022 (Adobe, San Jose, CA, USA).

## Results and Discussion

### Venom composition

**Figure 1:**
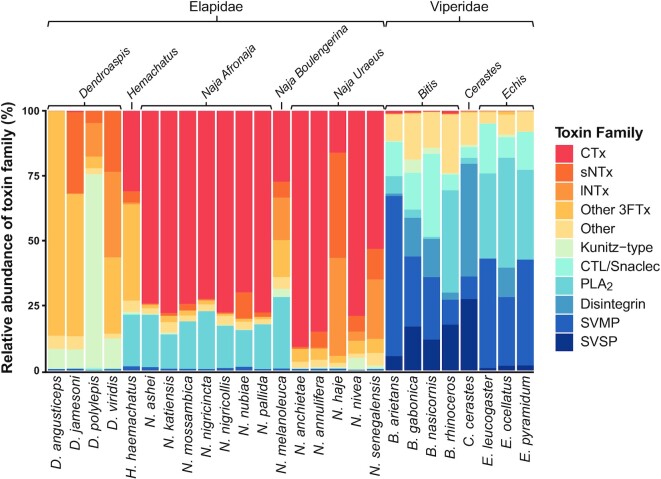
Composition of the whole venoms of the 26 medically most important elapids and vipers from sub-Saharan Africa. Toxins are grouped according to protein families and expressed as a percentage of total identified proteins (mol/mol). SVMP disintegrins have been classed as SVMPs, while non-SVMP disintegrins have been classed as disintegrins (as per UniProt definitions). CTL, C-type lectin; CTx, cytotoxin; lNTx, long neurotoxin; PLA_2_, phospholipase A_2_; sNTx, short neurotoxin; SVMP, snake venom metalloproteinase; SVSP, snake venom serine proteinase; 3FTx, 3-finger toxin.

The venom proteomes of the 26 medically most important elapids and vipers from sub-Saharan Africa (Table 2) were determined using a bottom-up proteomics approach, involving the enzymatic digestion of whole venoms, separation and analysis by LC-MS/MS, assignment of the identified proteins to their respective protein families, and calculation of protein family abundances as a percentage of total identified proteins (mol/mol) (Fig. 1, Supplementary Table S1). In addition, the RP-HPLC chromatograms of the venoms are shown in Supplementary Fig. S1.

**Table 2:** Functional *in vitro* activities of whole venoms from the 26 medically most relevant elapids and vipers in sub-Saharan Africa. Color scales indicate the IC_50_ and EC_50_ values. ND, not determined (response curves did not reach saturation); -, not tested.

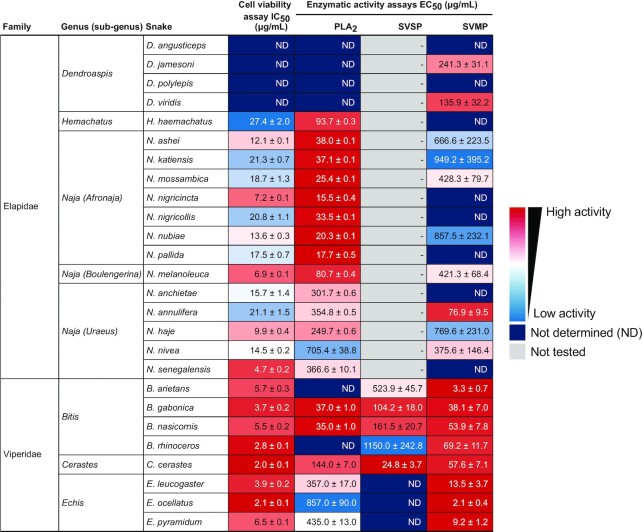

### Elapidae

The elapids included in this study belong to the genera *Naja,Hemachatus*, or *Dendroaspis* (Table 2). Of these, the true cobra lineage (*Naja* sp.) is by far the most widespread and diverse group throughout the African continent. To reflect their evolutionary and ecological diversity, African true cobras can be further divided into the 3 subgenera *Afronaja, Boulengerina*, and *Uraeus* [28].

The subgenus *Afronaja* includes all African spitting cobras (*Naja ashei, Naja katiensis, Naja mossambica, Naja nigricincta, Naja nigricollis, Naja nubiae*, and *Naja pallida*), with representative species found from Egypt (*N. nubiae*) to South Africa (*N. mossambica* and *N. nigricincta*) [29]. Despite the widespread distribution and different habitat preferences of *Afronaja* species, their toxin arsenal is remarkably conserved both inter- and intraspecifically [29]. In terms of protein abundance, the proteomic analysis shows that the bulk of their venom consists of 3FTxs (∼79%; of which 75% are CTxs) and PLA_2_s (∼17%), which is in accordance with previous studies [29, 30]. Notably, *N. nubiae* diverges from the general *Afronaja* venom profile and contains a considerable amount of short neurotoxins (sNTxs, ∼10%). Envenomings by *N. nubiae* therefore often result in both cytotoxic and neurotoxic clinical manifestations [30] compared to the mostly cytotoxic manifestations seen in envenomings caused by the rest of *Afronaja* species.

The only member of the subgenus *Boulengerina* included in this study was the forest cobra (*Naja melanoleuca*) [31], native to the forests and savannahs of central Africa, where it feeds on reptiles, amphibians, birds, small mammals, and even fish [32, 33]. Its venom was shown to have a high content of 3FTxs (∼64%), of which most were CTxs (∼27%), and also a considerable amount of PLA_2_s (∼28%), which is in agreement with a previous study [19]. Additionally, the venom contained a substantial proportion of long neurotoxins (lNTxs; ∼16%), the fourth highest among all snakes in this study, which could explain the neurotoxic manifestations reported after envenoming [34]. One toxin family in which the abundance differed from an earlier study was the SVMPs, where Lauridsen et al. [19] reported an abundance of 9.7%, whereas we only found 0.7%. Plausible explanations for this discrepancy could be a combination of variation between venom batches as a consequence of intraspecific venom variability, differences in the units used to quantify relative protein abundance (mol/mol in this study vs. wt% in Lauridsen et al. [19]), and/or the use of different proteomic methods (no decomplexing step prior to mass spectrometry in this study). Notably, the venom profile of *N. melanoleuca* differed from the other 2 *Naja* subgenera, containing less CTxs than *Afronaja* and more PLA_2_s than *Uraeus*.

The *Uraeus* subgenus (*Naja anchietae, Naja annulifera, Naja haje, Naja nivea*, and *Naja senegalensis*) consists of species with predominantly neurotoxic effects [34]. Like *Afronaja*, this lineage is widespread across the continent from Morocco (*N. haje*) to South Africa (*N. anchietae, N. annulifera*, and *N. nivea*) and has a highly diverse diet, which includes amphibians, reptiles, birds, and other snakes [35]. The venoms in this subgenus were predominantly composed of 3FTx (∼95%), most of which were CTxs as in the *Afronaja* subgenus, despite the mainly neurotoxic characteristics of *Uraeus* envenomings. This was not the case for *N. haje*, which contained mainly sNTxs (40%), lNTxs (38%), and CTxs (16%), although a previous study has reported a higher abundance of CTxs [36]. *N. senegalensis* also contained significant amounts of sNTxs (12%) and lNTxs (23%). Moreover, all *Uraeus* species except *N. anchietae* contained some neurotoxins (at least ∼6%), in line with the neurotoxic clinical manifestations associated with envenomings caused by these snakes. For snakes where proteomics data were available, our data corroborate the previously reported findings for *N. senegalensis* [37] and *N. nivea* [38]. For *N. annulifera* [39], a previous study reported an abundance 11.18% SVMPs, while our data showed only 0.6%, which, as mentioned previously, could be due to intraspecific venom variability and/or different methods for proteomics and quantification. In contrast to the other *Naja* species, *Uraeus* cobras all showed very low levels of PLA_2_s (∼0.15%), which were up until recently thought to be almost ubiquitous in snake venoms [40]. Of note, the proteomic venom composition for *N. anchietae* is presented here for the first time, strengthening a previous theory that low levels of PLA_2_s are a feature of all snakes within the *Uraeus* subgenus [37].

The rinkhals (*Hemachatus haemachatus*) is a spitting elapid classified in their own monotypic genus despite greatly resembling true cobras in morphology and general biology. This species is native to southeastern Africa, where it can be found in several ecosystems (e.g., savannah, woodland, and shrubland) and is known to prey mainly, although not exclusively, on amphibians [35]. According to our proteomic data, its venom composition is similar to those of the *Afronaja*, spitting cobras, which convergently evolved the ability to spit venom. In fact, *H. haemachatus* venom was shown to have a high content of 3FTxs (∼73%) and PLA_2_s (∼20%). Of the 3FTxs identified, the most abundant were CTxs or CTx homologs, with a small amount of sNTxs. This correlates with the cytotoxic and neurotoxic clinical manifestations of *H. haemachatus* envenomings [41]. The predominance of 3FTxs and PLA_2_s in this species is in line with a previous study by Sánchez et al. [8]. However, there are some minor discrepancies, as this previous study detected a larger amount of SVMPs (7% vs. 1.4%).

All 4 members of the *Dendroaspis* genus (mambas) were included in this study, namely, *Dendroaspis angusticeps, Dendroaspis polylepis, Dendroaspis jamesoni*, and *Dendroaspis viridis*. Widespread throughout the African continent, these species constitute one of the few predominately arboreal elapid lineages worldwide, cruising through the canopy of rainforest and woodland regions [42, 43]. As an exception, *D. polylepis* is often (but not always) more ground-dwelling than its congeners, being commonly found in open savannas and rocky hills [44, 45]. Overall, mambas mainly prey on birds and small mammals such as rodents and bats [42, 46], although their diet and ecology are poorly known.

Signature components of mamba venoms are the presynaptic neurotoxins called dendrotoxins, which belong to the Kunitz-type protease inhibitor family [12, 13]. Dendrotoxins are especially abundant in *D. polylepis* venom, where Kunitz-type protease inhibitors account for 75% of the venom proteins, as shown in this and previous studies [18, 47]. Conversely, *D. angusticeps* venom mainly consists of 3FTxs (87%; mainly short-chain aminergic and orphan group toxins), which is once again in accordance with previous findings [42]. The venoms of *D. jamesoni* and *D. viridis* are very similar when comparing the abundance of Kunitz-type protease inhibitors, sNTx, and other 3FTxs (Fig. 1, Supplementary Table S1), but different in terms of lNTx abundance (0.2% and 33%, respectively). Notably, our results are also in agreement with a recent study on venom gland transcriptomics for all 4 *Dendroaspis* species [17], indicating a general pattern of matching abundance profiles between venom transcriptome and proteome in mambas.

### Viperidae

The Viperidae family is represented by the genera *Bitis, Cerastes*, and *Echis* in this study. Of these, *Bitis* is the most geographically widespread and taxonomically diverse viperid genus in Africa, with 18 currently recognized species (commonly referred to as African adders) found from Morocco to South Africa [43]. More specifically, *B. rhinoceros* is found in western Africa from Guinea to Togo, while *B. gabonica* and *B. nasicornis* occur from Nigeria to central, eastern, and southern Africa [2]. Last, *B. arietans* occurs across open woodland, grassland, and semiarid habitats throughout sub-Saharan Africa, southern Arabia, and Morocco [48]. Large-sized African adders like those included in this study are mostly generalist predators feeding on small mammals, birds, lizards, and occasionally toads [49, 50].

The venom compositions of *B. gabonica* and *B. nasicornis* are rather similar and show a high abundance of SVMPs (27% and 24%), SVSPs (17% and 12%), disintegrins (15%), and C-type lectins (CTLs)/snaclecs (14% and 32%). SVMPs dominate the venom of *B. arietans* (62%), whereas *B. rhinoceros* venom is particularly rich in PLA_2_s (39%) and SVSPs (18%). All these 4 species have been analyzed previously by Calvete et al. [51], and overall, our data correlate relatively well with this previous study with some variations observed regarding PLA_2_s (*B. gabonica, B. nasicornis*, and *B. rhinoceros*), disintegrins (*B. arietans, B. gabonica*, and *B. nasicornis*), SVMPs (*B. rhinoceros*), and CTLs (*B. nasicornis*) (Fig. 1, Supplementary Table S1). This discrepancy can be due to several reasons, such as using different methods to generate and analyze the data, as well as intraspecific variation in venom composition [52]. Furthermore, the complexity of some snake venom components (e.g., SVMPs exist in a size range from 20 to 100 kDa [53]) may lead to inconsistencies between studies. This emphasizes the importance of using the same proteomic approach for cataloguing venom composition of different snakes to enable comparison. Large amounts of rhinocerase 2, a SVSP homolog that contains a H57R mutation [54], was found in the venom of *B. rhinoceros*. Interestingly, this H57R mutation was also found in peptides from *B. gabonica* and *B. nasicornis*, yet in smaller amounts. Such SVSP homologs have previously been detected in *B. gabonica* [55], but this is the first time they have been found in *B. nasicornis*.

The only member of the *Cerastes* genus included in this study is the Saharan horned viper (*Cerastes cerastes*). This species is distributed throughout North Africa and further eastward as far as southwestern Israel and southwestern Saudi Arabia [56]. Like many other viper lineages, *C. cerastes* is an ambush predator, relying on camouflage to lunge at small rodents and lizards by surprise [57]. Our proteomics analysis of *C. cerastes* venom shows a high abundance of SVSPs (27%) as opposed to a relatively low abundance of CTLs (4%), which is in agreement with other studies [58–60] (Fig. 1, Supplementary Table S1). On the other hand, the largest discrepancy compared to previous reports is observed for SVMPs, which were found to only constitute 8.6% of venom proteins in this study compared to 30–60% reported in literature, and disintegrins, which were found to constitute 43% of venom proteins in this study compared to approximately 10% previously reported in literature [58–60]. It is important to note that some SVMPs contain disintegrin (P-II class) or disintegrin-like (P-III class) domains [53], and therefore, mismapping of the peptides is possible but unlikely in this particular case since all such peptides mapped exactly to known *C. cerastes* disintegrins in the UniProt database.

The final Viperidae genus included in this study is *Echis*. The family members included are *E. ocellatus, E. pyramidum*, and *E. leucogaster*, of which no proteomic data have been reported previously for the latter. *E. ocellatus* and *E. pyramidum* are distributed throughout northern Africa, while *E. leucogaster* occurs in West Africa, isolated areas of the western Sahara, and throughout Algeria [61]. The diet of *Echis* snakes is widely varied, including invertebrates, such as scorpions and centipedes, small mammals, birds, lizards, and amphibians [62]. Our proteomics data show that the venom of *E. pyramidum* and *E. leucogaster* mainly consists of SVMPs with abundances of ∼41% and ∼42%, respectively. In contrast, *E. ocellatus* mainly consists of PLA_2_s with an abundance of ∼42%, followed by ∼26% SVMPs (Fig. 1, Supplementary Table S1). This differs from previous studies, where SVMPs were also reported as the major component of *E. ocellatus* venom with abundances of ∼70% [63, 64]. Again, different methods used to analyze the venom composition and the origin of the snakes milked to obtain the venoms may be underlying reasons for the observed differences. SVSPs of *Echis* venom comprise less than 2% of the whole venom (Fig. 1, Supplementary Table S1), which is in accordance with previous reports [63, 64]. In agreement with a previous study showing that the genetic variability between *E. pyramidum* and *E. leucogaster* is very low [65], our proteomics data show that the venom composition of *E. leucogaster* is quite similar to that of *E. pyramidum*. Strikingly, the amount of disintegrins was found to be 11% for *E. ocellatus* and less than 0.01% for *E. pyramidum* and *E. leucogaster* (Fig. 1, Supplementary Table S1). Extensive, likely diet-driven, interspecific venom variation has been documented in *Echis* representatives at the transcriptome and proteome levels [63] and in functional toxicity studies [66–68]. It is plausible that this interspecific variation can explain the differences between our results and previous analyses of *E. pyramidum* and *E. ocellatus* venoms.

### *In vitro* functional characterization of whole venoms

To evaluate and compare functional activities of sub-Saharan Africa's 26 medically most relevant elapid and viper venoms, we determined the concentrations of snake venom resulting in 50% product conversion at a fixed substrate concentration (EC_50_ values) in PLA_2_, SVSP, and SVMP enzymatic activity assays, as well as the concentrations of snake venom reducing the cell viability by 50% (IC_50_ values) in a cell viability assay. Lower EC_50_ or IC_50_ values indicate more potent activity of the analyzed toxins in the whole venom.

### PLA_2_ enzymatic activity

Secreted PLA_2_s are one of the major components of many animal venoms. These 13- to 15-kDa enzymes need Ca^2+^ ions to catalyze the hydrolysis of phospholipids [69]. However, it is noteworthy that some PLA_2_s have lost their enzymatic activity during evolution [70]. In the noncatalytic PLA_2_s, the catalytic residue D49 is mutated to another amino acid (e.g., lysine, serine, asparagine, glutamine, or arginine), resulting in a conformational change of the Ca^2+^ binding loop that prevents the reaction by hindering Ca^2+^ coordination, which is essential for catalysis [71, 72]. Despite sharing a 40–99% amino acid sequence identity and highly conserved 3-dimensional structures, snake venom PLA_2_s display a wide variety of pharmacologic activities, including neurotoxic, myotoxic, cytotoxic, anticoagulant, and hemolytic effects [73].

**Figure 2:**
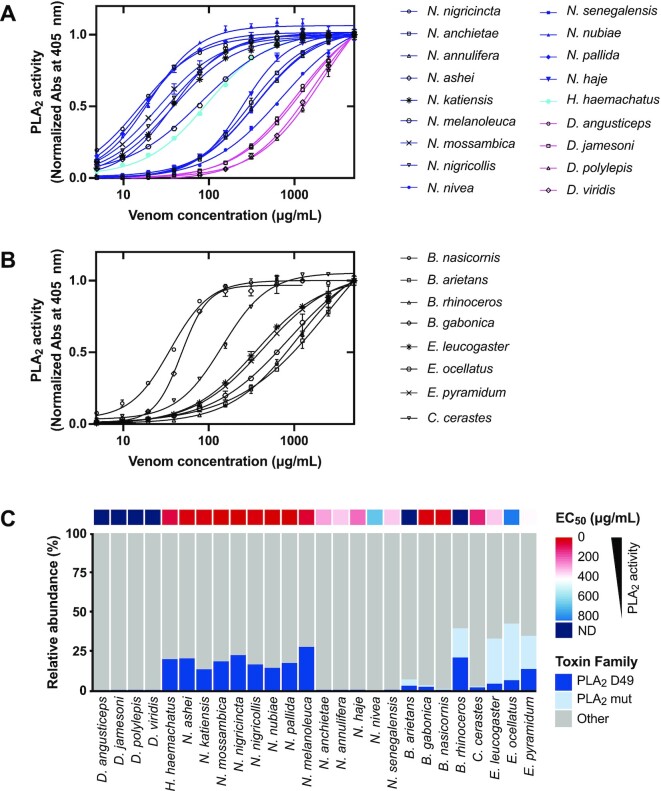
PLA_2_ enzymatic activity of whole venoms of elapids (A) and vipers (B) at different venom concentrations. Absorbances at 405 nm were normalized by subtracting values of the negative control (absence of venom). Error bars: SD from 2 independent measurements. (C) Relative abundance of enzymatically active PLA_2_s (D49) and enzymatically inactive PLA_2_s (mut) in 26 sub-Saharan snake venoms. A heatmap displaying EC_50_ values for each snake venom is plotted above the corresponding abundance.

Among the 18 snake species of the Elapidae family included in this study, the subgenus *Afronaja* shows the highest PLA_2_ activity with EC_50_ values of 15–38 µg/mL (Fig.   2A, Table 2). The subgenus *Boulengerina* and genus *Hemachatus* have moderate PLA_2_ activity, with EC_50_ values of 80–90 µg/mL, while the subgenus *Uraeus* exhibits low PLA_2_ activity with EC_50_ values above 200 µg/mL. Finally, *Dendroaspis* venoms display the weakest PLA_2_ activity (EC_50_ values over 1 mg/mL), which is in agreement with previous findings [18, 74]. The PLA_2_ activity of elapid snake venoms can therefore be ranked in the following order: *Afronaja > Boulengerina > Hemachatus* > *Uraeus > Dendroaspis*. This is in agreement with a previous publication on PLA_2_ activity of the 3 *Naja* subgenera [40] and correlates to the relative abundance of PLA_2_s in our proteomics data, except for *N. melanoleuca*, which exhibits the highest PLA_2_ abundance among the elapids but slightly lower activity than the 7 snakes from the *Afronaja* subgenus (Fig. 2A, Table 2).

Within the 8 snakes from the Viperidae family, *B. gabonica* and *B. nasicornis* show the lowest EC_50_ values for PLA_2_ activity (∼40 µg/mL; Fig.   2B, Table 2), which is comparable to that of the subgenus *Afronaja* from the Elapidae family_._ The high PLA_2_ activity of *B. gabonica* and *B. nasicornis* venom is in agreement with a previous publication [51] but differs from our proteomics data, which show a low PLA_2_ abundance (Fig. 2C). Notably, 2 other species from the *Bitis* genus, *B. arietans* and *B. rhinoceros*, show weak PLA_2_ activity, likely due to high abundances of mutated PLA_2_s in their venoms (Fig. 2C). Similarly, the 3 species from the *Echis* genus exhibit high relative abundances of PLA_2_ but weak enzymatic activity, which can be explained by high amounts of noncatalytic PLA_2_s found in *Echis* venoms (Fig. 2C). This result is consistent with a previous study reporting high myotoxic activity, but low enzymatic activity, of S49 PLA_2_s in *E. ocellatus* and *E. pyramidum* venoms [75]. The *Cerastes* genus displays the second highest PLA_2_ activity, with an EC_50_ value of 144 µg/mL. In general, our data demonstrate that there is a high correlation between PLA_2_ activity and PLA_2_ relative abundance for most of the viperid snake venoms.

### SVSP enzymatic activity

SVSPs are members of the S1 peptidase family, which catalyze the cleavage of covalent peptide bonds of proteins via the conserved catalytic triad H57–D102–S195, in which serine serves as the nucleophilic amino acid at the active site [76]. These 26- to 67-kDa enzymes affect the coagulation cascade, as well as the fibrinolytic and kallikrein–kinin systems, and cause hemostatic imbalances in victims [77]. In this relation, SVSPs can be classified as procoagulant, anticoagulant, platelet aggregating, or activator of fibrinolysis [78].

**Figure 3:**
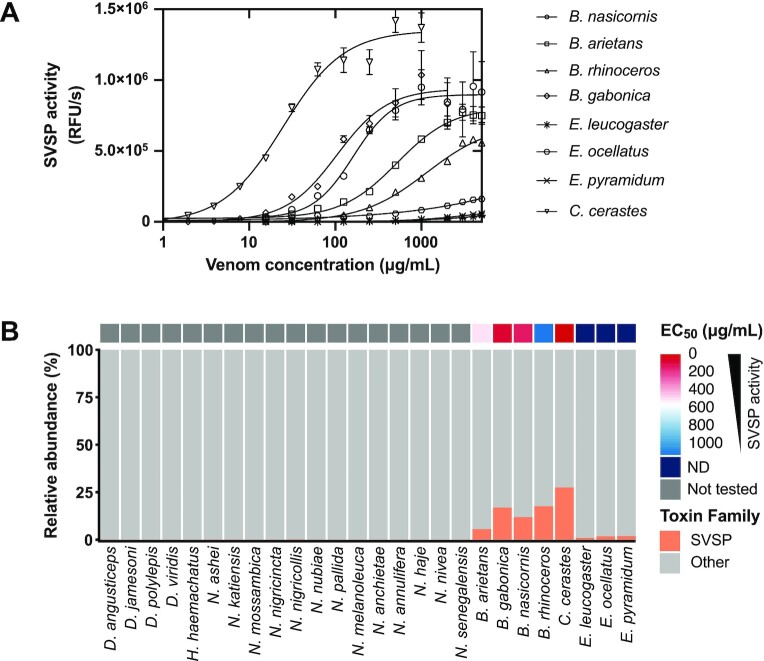
(A) SVSP enzymatic activity of viper whole venoms at different venom concentrations. RFU, relative fluorescence unit. Error bars: SD from 2 independent measurements. (B) Relative abundance of SVSPs in 26 sub-Saharan snake venoms. A heatmap displaying EC_50_ values for each snake venom is plotted above the corresponding abundance.

Our proteomic data show negligible amounts of SVSPs in the elapid venoms (Fig. 3B), and therefore, EC_50_ values of SVSPs were determined only for viperid venoms (Fig. 3A, Table 2). *C. cerastes* shows the lowest EC_50_ value (25 µg/mL), which correlates with the high SVSP abundance in its venom (27.5%, Fig.   3B). Within the genus *Bitis*, the SVSP activity of *B. gabonica* and *B. nasicornis* is moderate, with EC_50_ values of ∼100 µg/mL, whereas *B. arietans* and *B. rhinoceros* demonstrate weak SVSP activity with EC_50_ values between 500 and 1,000 µg/mL (Fig.   3A, Table 2). Although *B. rhinoceros* exhibits the second highest SVSP abundance among the 8 vipers included in this study, its EC_50_ value is in the high range. This could be because, as mentioned before, an SVSP homolog with a catalytic site mutation [ 54] was found in our proteomic analysis. Finally, the genus *Echis* shows the weakest SVSP activity, with EC_50_ values above 1 mg/mL, which agrees with their low SVSP abundance (below 2%, Fig. 3B).

### SVMP enzymatic activity

The Zn^2+^-dependent SVMPs are one of the most abundant toxins in viperid venoms [5] mainly responsible for inducing systemic hemorrhage after envenomings with these snakes. There are 3 major classes of SVMPs: P-I contains only a metalloproteinase (M) domain, P-II contains an M domain and a disintegrin (D) domain, and the most complex P-III class is composed of an M domain, a D domain, and a cysteine-rich (C) domain.

**Figure 4:**
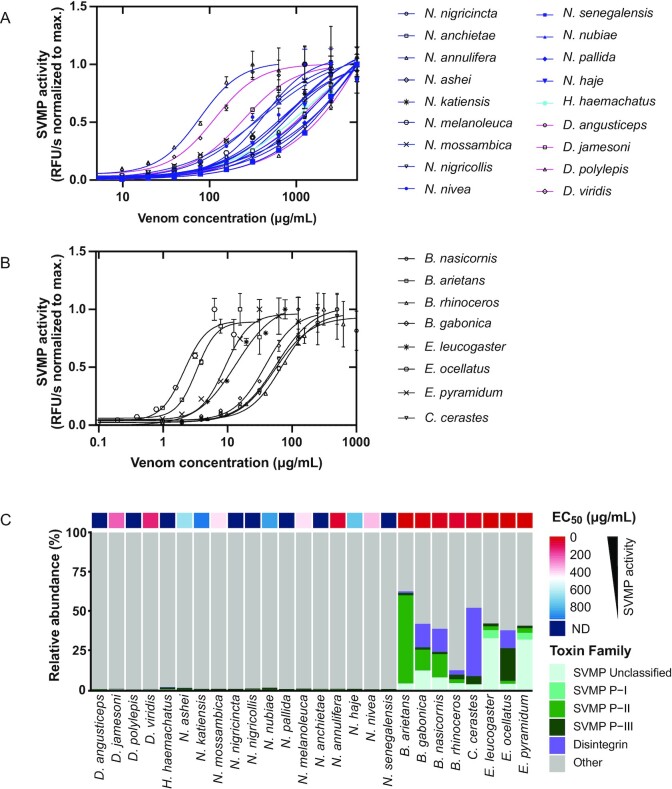
SVMP enzymatic activity of the whole venoms of elapids (A) and vipers (B) at different venom concentrations. RFU, relative fluorescence unit. Error bars: SD from 2 independent measurements. (C) Relative abundance of SVMP subfamilies in 26 sub-Saharan snake venoms. SVMP disintegrins are included in the SVMP category. A heatmap displaying EC_50_ values for each snake venom is plotted above the corresponding abundance.

All elapids show high EC_50_ values of at least 100 µg/mL, except *N. annulifera*, with a value of 80 µg/mL. This is in agreement with previously published data showing that SVMP is the second most abundant protein family in *N. annulifera* venom after 3FTxs [39]. SVMPs in the *Dendroaspis* species have a low abundance but a high activity in *D. jamesoni* and *D. viridis* (EC_50_ values of 241 and 136 µg/mL, respectively). This is in agreement with an earlier study in which SVMP-dependent anticoagulant activity was observed in *Dendroaspis* species despite the low SVMP abundance [79]. Among the 8 viperids included in this study, *B. arietans* show the lowest EC_50_ value (3.3 µg/mL), while the 3 venoms from the *Echis* subgenus show high amounts of SVMPs (Fig. 4C), with EC_50_ values between ∼2 and 14 µg/mL (Fig.   4B, Table 2). *B. arietans* has an EC_50_ value in the same range as the *Echis* venom, whereas all other *Bitis* species have EC_50_ values around 40–50 µg/mL.

### Cell viability

CTxs and PLA_2_s are known to, either individually or synergistically, interfere with and disrupt the integrity of cellular membranes, leading to irreversible damage and cell death [25, 80]. In snake venoms, cytotoxins are mainly found in the genera *Naja* and *Hemachatus* of the Elapidae family [8], while PLA_2_s are found in all venomous snake families, including Elapidae and Viperidae [81]. Therefore, the cytotoxicity of all elapid and viperid venoms included in this study was evaluated using an immortalized human keratinocyte cell line, which has been reported to be sensitive to snake venom cytotoxins and PLA_2_s [25].

**Figure 5:**
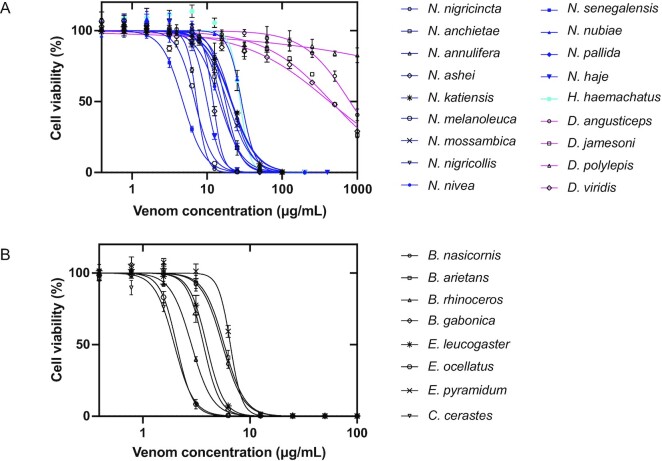
Cell viability of the N/TERT keratinocyte cell line after addition of different concentrations of whole venoms of elapids (A) and vipers (B). The negative control value (without venom) was set to 100%. Error bars: SD from 2 independent measurements.

Treatment of the cells with venoms resulted in a concentration-dependent inhibition of cell viability (Fig. 5). As expected, the viper venoms were more potent (IC_50_ 2.0–6.5 µg/mL) than venoms from the elapids (IC_50_ 4.7 to >100 µg/mL). Among the Elapidae, 4 species from the *Naja* genus (i.e., *N. senegalensis, N. melanoleuca, N. nigricincta*, and *N. haje*) show IC_50_ values close to those of the Viperidae (below 10 µg/mL), while the *Dendroaspis* genus demonstrates IC_50_ values above 100 µg/mL. These results are in alignment with a high abundance of cytotoxins in the *Naja* genus and PLA_2_s in the Viperidae family, as well as a very low abundance of these 2 toxin families in the *Dendroaspis* genus.

### Thromboelastography

The blood coagulation cascade is a primary target for many snake venom toxins due to its pivotal role in maintaining homeostasis, and most major venomous snake families possess toxins in their venoms that can interfere with this system. This is particularly evident in (although not exclusive to) vipers, whose venoms are generally dominated by proteins that cause coagulopathies (e.g., SVMPs, SVSPs, and disintegrins) [82, 83]. Thus, we assessed the coagulotoxic effects of the venoms of all viper species included in this study via TEG by incubating whole venom with human plasma and physiologic cofactors of coagulation (i.e., calcium and phospholipids).

**Figure 6:**
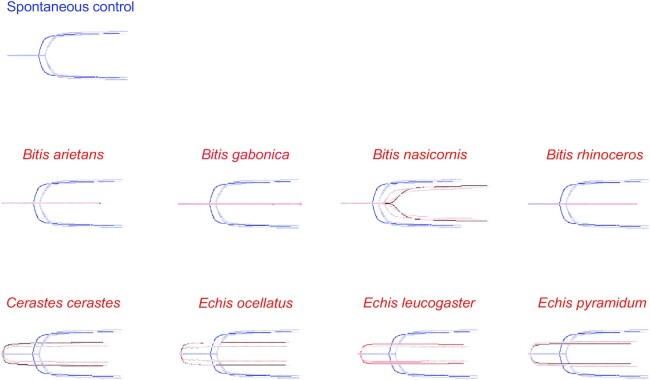
Overlaid thromboelastography traces showing the ability of the different venoms (2 µg/mL for all species except *B. arietans*, 20 µg/mL) to clot plasma relative to a spontaneous control in 1 hour. Time is plotted horizontally and amplitude (clotting strength) is plotted vertically. Eight representatives of the Viperidae family, which possess procoagulant (*C. cerastes, E. leucogaster, E. ocellatus*, and *E. pyramidum*) and anticoagulant (*B. arietans, B. gabonica,B. nasicornis*, and *B. rhinoceros*) venom, are depicted. Blue traces represent spontaneous clot controls and red traces represent samples (*n* = 3).

All venoms were tested and presented similar activity, at ∼2 µg/mL and ∼20 µg/mL, except *B. arietans*, which showed inconclusive results at ∼2 µg/mL. Both anti- and procoagulant effects were observed on a broadly genus-specific basis. More specifically, venoms from all *Bitis* species, except *B. nasicornis*, displayed an overall strong anticoagulant activity, with no visible clot formation (Fig. 6).

Unlike *Bitis, C. cerastes* venom produced a detectable, stable clot almost immediately after assay initiation (Fig. 6). Interestingly, other researchers have reported that the venom of this species exerts both pro- and anticoagulant effects in a concentration-dependent manner, whereby low venom concentrations (≤200 µg/mL), as used in our study, enhanced blood clotting, in agreement with our observations, while higher amounts disrupted coagulation [84].

Last, an even stronger procoagulant activity than seen for *C. cerastes* was observed for the 3 *Echis* representatives included in this study (Fig. 6). This is not surprising, as a signature trait of most *Echis* species is the presence of exceptionally potent prothrombin activators (all part of the SVMP family) in their venoms, which results in uncontrolled formation of fibrin clots due to excessive production of thrombin [85–88]. This agrees with our proteomic data, in which *Echis* venoms show high amounts of SVMPs for all 3 species.

## Conclusion

In this study, we systematically analyzed and compared the proteomics and *in vitro* functional activity of multiple snake venoms and provide toxicovenomic profiles of 26 of sub-Saharan Africa's medically most important elapids and vipers. To the best of our knowledge, the venom composition of *N. anchietae* and *E. leucogaster* is presented here for the first time. Overall, our data show that the elapid venoms contained large amounts of neurotoxic and cytotoxic 3FTxs and PLA_2_s, whereas the viper venoms were dominated by hemotoxic and/or cytotoxic PLA_2_s, SVMPs, and SVSPs, as expected based on clinical manifestations observed for elapid and viperid envenoming [5].

The high-throughput, label-free, quantitative proteomics approach presented here comes with some limitations. During mass spectrometry, proteins are identified through mapping of the peptide sequence to a database. It is important to keep in mind that peptides from highly similar isoforms may be difficult to map back to their parent proteins, resulting in false positives (e.g., detection of a disintegrin instead of an SVMP). Additionally, the lack of a comprehensive database may thus result in false negatives; if the sequence of a protein is not present in the database, it cannot be detected. In the UniProt reference database used here, there was a noticeable lack of SVMPs from the 26 snakes, apart from *B. gabonica,E. ocellatus*, and *E. pyramidum leakeyi* (a subspecies of *E. pyramidum*), where transcriptomic data were available. The NCBI protein database, a possible alternative database, had the same issue. Importantly, by including not only manually reviewed Swiss-Prot proteins in the UniProt database, we increased the number of SVMPs being identified. Interestingly, low amounts of SVMPs have been observed in previous studies using similar workflows [20]. Together, this indicates a clear need for further work to characterize SVMPs from medically relevant sub-Saharan African snake species.

With respect to functional activity, the subgenus *Afronaja*, together with *B. gabonica* and *B. nasicornis*, showed the highest enzymatic PLA_2_ activity, which highlights the importance of catalytic PLA_2_s in relation to the clinical manifestations observed after envenoming with these snakes, such as hemolytic and anticoagulant effects [89, 90]. When comparing the enzymatic SVSP activity among the vipers, the abundance of active SVSPs in the venoms showed a good correlation with activity; for the outlier *B. rhinoceros*, the high abundance but low activity of SVSPs can be explained by the high proportion of catalytically inactive SVSP homologs in its venom. All viper venoms were shown to possess high SVMP activity with low EC_50_ values, 10–100 times higher activity than most of the elapid venoms, where only *N. annulifera* venom showed activity in the same range as the vipers. Given that *N. annulifera* has been shown in a previous study to possess a substantial amount of SVMP in its venom [39], higher than many other cobra species, this is not too surprising.

The coagulotoxic effect of the viper venoms included in this study was assessed by TEG, showing a procoagulant effect for venoms from *C. cerastes* and the genus *Echis*. In contrast, all *Bitis* species showed strong anticoagulant activity except *B. nasicornis*, which also showed an anticoagulant activity but to a lesser extent. Finally, cell viability of a keratinocyte cell line was inhibited by addition of snake venoms from all snake species, except the ones from the genus of *Dendroaspis*. This is not surprising, given that *Dendroaspis* venoms are known to be highly neurotoxic, cause very little tissue damage, and display very low enzymatic activities [18, 74].

Some inconsistencies were observed between the relative abundance of certain toxin families and whole venom activity in their respective *in vitro* functional assays. For example, our data show that the venoms of *N. annulifera, D. viridis*, and *D. jamesoni* had high SVMP activity despite low abundance of such proteins. When it comes to *in vitro* assays for characterizing toxin functions, a limitation of the present study is the lack of assays to assess the activity of neurotoxins, as several species included herein (e.g., mambas and most *Uraeus* cobras) possess predominately neurotoxic venoms [18, 34, 74].

Overall, this study provides a foundation for further studies on snake biology and evolution, for which we recommend an integrated approach combining genomics, transcriptomics, and proteomics to provide information on gene expression and other molecular mechanisms linked to phenotypic diversity [1]. Moreover, the toxicovenomic profiles elucidated in this study may aid in the development of effective antivenoms through better understanding of the behavior of snake venoms and their roles as drug targets.

## Additional Files

**Supplementary Fig. S1**. RP-HPLC chromatograms of the whole venoms of 26 sub-Saharan snakes.

**Supplementary Table S1**. Composition of the whole venoms of 26 sub-Saharan snakes.

giac121_GIGA-D-22-00205_Original_Submission

giac121_GIGA-D-22-00205_Revision_1

giac121_Response_to_Reviewer_Comments_Original_Submission

giac121_Reviewer_1_Report_Original_Submission Michael Hogan -- 8/30/2022 Reviewed

giac121_Reviewer_1_Report_Revision_1 Michael Hogan -- 10/13/2022 Reviewed

giac121_Reviewer_2_Report_Original_Submission Benjamin-Florian Hempel -- 9/13/2022 Reviewed

giac121_Supplemental_Figure_and_Table

## Data Accessibility

The data sets supporting the results of this article, including results from the *in vitro* functional activity assays, are available in the *GigaScience* GigaDB repository [92]. Results from the proteomics characterizations have been deposited to the ProteomeXchange Consortium via the PRIDE [91] partner repository with the dataset identifier PXD036161.

## Abbreviations

CTLs: C-type lectins; CTxs: cytotoxins; FA: formic acid; LC: liquid chromatography; lNTxs: long neurotoxins; MS: mass spectrometry; NOB: 4-nitro-3-(octanoyloxy)benzoic acid; PBS: phosphate-buffered saline; PLA_2_s: phospholipase A_2_s; RFU/s: relative fluorescence units per second; RP-HPLC: reversed-phase high-performance liquid chromatography; sNTxs: short neurotoxins; SVMPs: snake venom metalloproteinases; SVSPs: snake venom serine proteinases; 3FTxs: 3-finger toxins; TEG: thromboelastography; TFA: trifluoroacetic acid.

## Competing Interests

All authors declare no conflict of interest.

## Funding

This research was funded by a grant from Wellcome (221702/z/20/z).

## Authors’ Contributions

G.T.T.N., C.O.B., A.H.L., and A.L. conceived the study. G.T.T.N., C.O.B., Y.W., L.S., A.G.C., and I.C.P. performed laboratory experiments and analyzed the data. A.H.L. and A.L. supervised the study. G.T.T.N., C.O.B., Y.W., L.S., A.G.C., S.A., A.H.L., and A.L. drafted the manuscript. All authors read and approved the final manuscript.
