# Supplementary material for: High-throughput proteomics and in vitro functional characterization of the 26 medically most important elapids and vipers from sub-Saharan Africa
Source: Gigascience. 2022 Dec 13;11:giac121. doi: 10.1093/gigascience/giac121 (PMC9744630; doi:10.1093/gigascience/giac121)
Supplement: giac121_GIGA-D-22-00205_Original_Submission [file giac121_giga-d-22-00205_original_submission.pdf]

GigaScience

High-throughput proteomics and in vitro functional characterization of the 26 medically most important elapids and vipers from sub-Saharan Africa

--Manuscript Draft--

**Manuscript Number:**	GIGA-D-22-00205
**Full Title:**	High-throughput proteomics and in vitro functional characterization of the 26 medically most important elapids and vipers from sub-Saharan Africa
**Article Type:**	Research
**Funding Information:**	Wellcome Trust (221702/z/20/z) — Prof Andreas H. Laustsen
**Abstract:**	Venomous snakes are important parts of the ecosystem, and their behavior and evolution have been shaped by their surrounding environments over the eons. This is reflected in their venoms, which are typically highly adapted for their biological niche, including their diet and defense mechanisms for deterring predators. Sub-Saharan Africa is rich in venomous snake species, of which many are dangerous to humans due to the high toxicity of their venoms and their ability to effectively deliver large amounts of venom into their victims via their bite. In this study, the venoms of 26 of sub-Saharan Africa's medically most relevant elapid and viper species were subjected to parallelized toxicovenomics analysis. The analysis included venom proteomics and in vitro functional characterization of whole venom toxicities and enables a robust comparison of venom profiles between species. The data presented here corroborates previous studies and provides biochemical details for the clinical manifestations observed in envenomings by the 26 snake species. Moreover, two new venom proteomes (N. anchietae and E. leucogaster) are presented here for the first time. Combined, the presented data can help shine light on snake venom evolutionary trends and possibly be used to further improve or develop novel antivenoms.
**Corresponding Author:**	Andreas Hougaard Laustsen<br>Technical University of Denmark<br>Kongens Lyngby, DENMARK
**Corresponding Author Secondary Information:**	
**Corresponding Author's Institution:**	Technical University of Denmark
**Corresponding Author's Secondary Institution:**	
**First Author:**	Giang Thi Tuyet Nguyen
**First Author Secondary Information:**	
**Order of Authors:**	Giang Thi Tuyet Nguyen
	Carol O'Brien
	Yessica Wouters
	Lorenzo Seneci
	Alex Gallissà Calzado
	Isabel Campos Pinto
	Shirin Ahmadi
	Andreas H. Laustsen
	Anne Ljungars
**Order of Authors Secondary Information:**	
**Additional Information:**	
**Question**	**Response**

Powered by Editorial Manager® and ProduXion Manager® from Aries Systems Corporation

Are you submitting this manuscript to a special series or article collection?	No
**Experimental design and statistics**<br><br>Full details of the experimental design and statistical methods used should be given in the Methods section, as detailed in our Minimum Standards Reporting Checklist. Information essential to interpreting the data presented should be made available in the figure legends.<br><br>Have you included all the information requested in your manuscript?	Yes
**Resources**<br><br>A description of all resources used, including antibodies, cell lines, animals and software tools, with enough information to allow them to be uniquely identified, should be included in the Methods section. Authors are strongly encouraged to cite Research Resource Identifiers (RRIDs) for antibodies, model organisms and tools, where possible.<br><br>Have you included the information requested as detailed in our Minimum Standards Reporting Checklist?	Yes
**Availability of data and materials**<br><br>All datasets and code on which the conclusions of the paper rely must be either included in your submission or deposited in publicly available repositories (where available and ethically appropriate), referencing such data using a unique identifier in the references and in the "Availability of Data and Materials" section of your manuscript.<br><br>Have you have met the above requirement as detailed in our Minimum	Yes

Standards Reporting Checklist?	

Powered by Editorial Manager® and ProduXion Manager® from Aries Systems Corporation

Click here to access/download;Manuscript;Nguyen et al. Submission GigaScience.docx

High-throughput proteomics and *in vitro* functional characterization of the 26 medically most important elapids and vipers from sub-Saharan Africa

Giang Thi Tuyet Nguyen[1¶], Carol O'Brien[1¶], Yessica Wouters[1], Lorenzo Seneci[1], Alex Gallissà Calzado[1], Isabel Campos Pinto[1], Shirin Ahmadi[1], Andreas H. Laustsen[1]*, Anne Ljungars[1]

[1]Department of Biotechnology and Biomedicine, Technical University of Denmark, DK-2800 Kongens Lyngby, Denmark

¶ These authors contributed equally

*Corresponding author:

Andreas Hougaard Laustsen; ahola@bio.dtu.dk; Tel.: +45-2988-1134

Abstract:

Venomous snakes are important parts of the ecosystem, and their behavior and evolution have been shaped by their surrounding environments over the eons. This is reflected in their venoms, which are typically highly adapted for their biological niche, including their diet and defense mechanisms for deterring predators. Sub-Saharan Africa is rich in venomous snake species, of which many are dangerous to humans due to the high toxicity of their venoms and their ability to effectively deliver large amounts of venom into their victims via their bite. In this study, the venoms of 26 of sub-Saharan Africa's medically most relevant elapid and viper species were subjected to parallelized toxicovenomics analysis. The analysis included venom proteomics and *in vitro* functional characterization of whole venom toxicities and enables a robust comparison of venom profiles between species. The data presented here corroborates previous studies and provides biochemical details for the clinical manifestations observed in envenomings by the 26 snake species. Moreover, two new venom proteomes (*N. anchietae* and

E. leucogaster) are presented here for the first time. Combined, the presented data can help shine light on snake venom evolutionary trends and possibly be used to further improve or develop novel antivenoms.

Keywords: Snakebite envenoming; sub-Saharan Africa; Toxicovenomic; *In vitro* venom characterization; High-throughput assays; Cytotoxicity; Enzymatic activity of venoms

Introduction

In the deep jungles, on the open savanna, and across deserts, snakes are omnipresent in sub-Saharan Africa, where they play an integral role in the natural ecosystems to which they have adapted over the course of evolution [1]. Some of these snake species are highly venomous, being classified by the World Health Organization as a category 1 or 2 snakes of the highest medical importance [2,3]. Thus understanding the composition and function of their venoms is not only important for elucidating basic biology and adaptation of species, but also of medical significance. Each year, venomous snakes inflict approximately 500,000 bites in Africa [4], causing major disability and disablement for many rural workers and children [5]. This challenge remains a pressing healthcare issue, which is further exacerbated by the socioeconomic impact that disability causes for manual laborers [6].

The medically most important snakes of sub-Saharan Africa belong mainly to the Elapidae (*e.g.* cobras, mambas, and rinkhals) and Viperidae families, although a few species from the Colubridae family (*e.g.* boomslang, *Dispholidus typus*) are also known to cause severe envenomings. Victims envenomed by elapid snakes typically display local as well as systemic clinical manifestations. Local manifestation often includes swelling, blistering, and bruising at the anatomical site of the bite, which may evolve into irreversible tissue necrosis and gangrene [7,8]. In comparison, systemic manifestations may include muscle twitching, spasms, weakness, fatigue, sleepiness, slurred speech, or difficulties to swallow. These can progress to flaccid paralysis and, in severe cases, fatal respiratory failure, unless mechanical ventilation is provided [7,9].

Similarly to elapids, envenomings caused by vipers may also result in both local and systemic manifestations. The victims often immediately feel a strong irradiating pain at the site of bite, and typically show hot inflammatory erythema, blisters, bruises, and

spontaneous bleeding [7]. Systemic clinical manifestations can include temporary loss of vision, fainting, and systemic hemorrhage, which in severe cases can lead to cardiovascular shock [7].

Different toxin families are responsible for the clinical manifestations observed for viper and elapid envenoming. As a first example, venom from spitting cobras is rich in cytotoxins (CTxs) from the three-finger toxin (3FTx) family and phospholipase A_2s (PLA_2s) [10], which interfere with and disrupt the integrity of cellular membranes, resulting in irreversible damage and cell death. In comparison, short and long-chain α-neurotoxins, another type of 3FTx, found in venoms such as those of the black mamba and forest cobra, block neuromuscular signaling and prevent normal muscle contractions through binding to acetylcholine receptors (nAChRs) on neuromuscular junctions [11]. Another example of a class of toxins that interfere with neuromuscular signaling are dendrotoxins. Dendrotoxins belong to the Kunitz-type inhibitors, are exclusively found in the venoms of mambas, and block ion transport through potassium channels, resulting in involuntary muscle contractions [12,13]. Snake venoms from most viperid species possess a high fraction of PLA_2s (*e.g.* Gaboon viper (*Bitis gabonica*)), snake venom metalloproteinases (SVMPs) (*e.g.* carpet viper (*Echis ocellatus*)), and snake venom serine proteinases (SVSPs) (*e.g.* horned viper (*Cerastes cerastes*)), which all play an important role in the toxicity of these venoms. SVMPs hydrolyze components of the cell wall of capillaries, which first reduce the mechanical integrity, and then disrupt the capillary walls, resulting in both local and systemic bleeding [14,15]. Systemic bleeding can also be caused by SVSPs that interfere with the blood coagulation cascade by decreasing the level of platelets, fibrinogen, and clotting factors [7,16].

So far, most venomic studies on African snakes have included only one or a small handful of species [17–21], and the inclusion of functional data has been somewhat

sporadic or absent. These studies have undoubtedly been important for obtaining a first snapshot of venom compositions, which have already enabled further studies within venom evolution, snake biology, and development of (recombinant) antivenom. However, the fact that these studies have been performed in multiple different laboratories using different protocols results in a limited level of data harmonization. To this end, and to elucidate a few so far undescribed venom proteomes, we describe high-throughput methods for proteomics (*i.e.*, venomics) and *in vitro* functional characterization of snake venoms (*i.e.*, toxicovenomics) in a parallelized manner and characterize sub-Saharan Africa's 26 medically most important snakes, comprising 18 elapids and 8 vipers (Table 1).

Material and Methods

Venoms and Reagents

Chemicals were obtained from Sigma-Aldrich (USA) unless otherwise stated. Venoms from the 26 snakes in Table 1 were obtained from Latoxan (France). PLA_2 substrate 4-nitro-3-(octanoyloxy)benzoic acid (NOB) and SVMP substrate ES010 were purchased from Enzo Life Sciences (USA). SVSP substrate (*p*-tosyl-Gly-Pro-Arg)$_2$-R110 and 96-well plates were purchased from Thermo Fisher Scientific (USA). All substrates for enzymatic assays were dissolved in DMSO to the stock concentration of 100 mM. CellTiter-Glo 3D Cell Viability Assay kit was obtained from Promega (USA).

Family	Genus (sub-genus)	Snake	Catalogue Number	Origin
Elapidae	*Dendroaspis*	*D. angusticeps*	L1307	Tanzania
		D. jamesoni	L1308	Cameroon
		D. polylepis	L1309	Kenya, South Africa
		D. viridis	L1310	Ghana
	Hemachatus	*H. haemachatus*	L1311	South Africa
	Naja (Afronaja)	*N. ashei*	L1375	Kenya
		N. katiensis	L1317	Burkina Faso

		N. mossambica	L1376	South Africa, Tanzania
		N. nigricincta	L1368	South Africa
		N. nigricollis	L1327	Cameroon, Tanzania, West Africa
		N. nubiae	L1342	Egypt
		N. pallida	L1321	Kenya
	Naja (Boulangerina)	*N. melanoleuca*	L1318	Cameroon, Ghana, Uganda
	Naja (Uraeus)	*N. anchietae*	L1374	Namibia
		N. annulifera	L1314	Sub-Saharan Africa
		N. haje	L1315	Egypt, Mali
		N. nivea	L1328	South Africa
		N. senegalensis	L1350	Mali
Viperidae	*Bitis*	*B. arietans*	L1159	Cameroon, Kenya, Mali, Saudi Arabia, West Africa, Tanzania
		B. gabonica	L1104	Burundi, Tanzania
		B. nasicornis	L1106	West Africa, Burundi
		B. rhinoceros	L1105	Ghana
	Cerastes	*C. cerastes*	L1107	Egypt, Tunisia
	Echis	*E. leucogaster*	L1109	Mali
		E. ocellatus	L1114	Cameroon, Mali, Ghana
		E. pyramidum	L1110	Egypt

Table 1. List of the 26 venoms from the medically most relevant elapids and vipers from sub-Saharan Africa used in this study. Catalogue number and origin are listed.

Venom fractionation by Reversed-Phase High-Performance Liquid Chromatography (RP-HPLC)

The venoms were separated by RP-HPLC using an Agilent Infinity II as previously described [22]. Briefly, lyophilized venom (10 mg) was dissolved in 1 mL of water containing 0.1% trifluoroacetic acid (TFA; solution A), centrifuged at 14,000 × *g* for 10 min, and transferred to a HPLC vial. For each fractionation round, 100 uL of sample was injected into an RP-HPLC C18 column (250 × 4.6 mm, 5 µm particle size) and eluted at 1 mL/min by applying a gradient towards acetonitrile containing 0.1% TFA (solution B) (0–15% B for 10 min, 15–45% B for 60 min, 45–70% B for 10 min, and 70% B for 9 min). Automatically collected fractions were dried using a vacuum centrifuge, resuspended in PBS, and stored at -80 °C.

Proteomic characterization of whole venom by mass spectrometry

In-solution tryptic digestion of the venom proteins

For each of the 26 snake venoms listed in Table 1, the lyophilized whole venom was dissolved in 1×PBS, then 5 µg was vacuum dried and resuspended in 20 µL of 6 M guanidinium hydrochloride containing 10 mM TCEP, 40 mM 2-Chloroacetamide, and 50 mM HEPES pH 8.5. After adding 40 µL of digestion buffer (10% acetonitrile, 50 mM HEPES pH8.5), samples were digested with LysC endopeptidase (1:50; w:w) for 3 h 30 min at 37 °C. Samples were further diluted with 140 µL of digestion buffer and mixed with trypsin (1:100; w:w). Trypsinized samples were incubated overnight at 37 °C, then diluted with 200 µL of 2% TFA to quench trypsin activity. Peptides were desalted on StageTip containing Empore C18 disks, eluted in 60 µL 40% acetonitrile containing 0.1% formic acid (FA), dried in a vacuum centrifuge, and resuspended in 2% acetonitrile containing 1% TFA and iRT peptides (Biognosys, Switzerland).

LC-MS/MS analysis

Mass spectrometry data was collected using a Q Exactive mass spectrometer (ThermoFisher Scientific, USA) coupled to a Thermo EASY-nLC 1200 liquid chromatography (LC) system (ThermoFisher Scientific). 100 ng of peptides were loaded into a 2 cm C18 trap column (ThermoFisher, 164705) connected to a 15 cm reverse-phase analytical column (ThermoFisher Scientific, ES900). Peptides were separated for 70 min with a gradient going from 10% to 60% buffer B (80% acetonitrile, 0.1% FA) over 60 min, until spiking to 95% buffer B for the last 10 min to wash the column. Full MS spectra were collected at a resolution of 70,000, with an AGC target of 3×10^6 or maximum injection time of 20 ms and a scan range of 300–1,750 m/z. The MS2 spectra were obtained at a resolution of 17,500, with an AGC

target value of 1×10^6 or maximum injection time of 60 ms, a normalized collision energy of 25 and an intensity threshold of 1.7×10^4. Dynamic exclusion was set to 60 s, and ions with a charge state < 2 or unassigned were excluded.

Using proteome Discoverer 2.4, peptide fragmentation spectra (MS/MS) were searched against a database consisting of all Swiss-Prot and TrEMBL protein sequences from the serpentes suborder available in Uniprot (331,759 entries). The search was performed using the built-in Sequest HT algorithm which was configured to derive fully-tryptic peptides using default settings. Cysteine carbamidomethyl was set as a static modification and oxidation (M), deamidation (N, Q) and acetyl on protein N-termini were set as dynamic modifications. Label-free quantitation was enabled in both processing and consensus steps, with quantitation being done using Minora Feature Detector. All results were filtered at 1% FDR and relative protein abundances were estimated by calculating the ratio of the of individual protein abundances to the sum of abundances of all proteins detected within a sample.

In vitro functional characterization of the whole venoms

PLA_2 enzymatic activity assay

The endpoint PLA_2 activity assay was run as described previously [23]. The snake venoms were dissolved at a concentration of 10 mg/mL in assay buffer (10 mM Tris pH 8, 100 mM NaCl, and 10 mM $CaCl_2$) and a 2-fold serial dilution (10 steps) was prepared. 100 µL/well of each dilution was added to a 96-well plate, together with 100 µL/well of NOB (final concentration 0.25 mM). The plates were shaken at 300 rpm for 2 min and then incubated at 37 °C for 40 min. The plates were then centrifuged (3000 × *g*, 4 °C, 3 min) before the absorbance was recorded at 405 nm using a VICTOR Nivo plate reader (Perkin Elmer, USA) at 25 °C. All reactions were run in duplicates and the absorbance values were shown as averages after subtracting a blank control containing no venom. EC_{50} values (the venom

concentration inducing half of the maximum absorbance at 405 nm proportional to product conversion) were determined using non-linear fitting with sigmoidal dose-response equation of the venom dose curves analyzed by GraphPad Prism 9 software (GraphPad Software Inc, USA).

SVSP and SVMP enzymatic activity assay

To measure SVSP and SVMP activities, enzymatic assays were set up. The hydrolysis reactions were performed in 96-well plates with a final volume of 100 µL per well. The snake venoms were dissolved in PBS for SVSP or assay buffer (10 mM Tris pH 8, 100 mM NaCl, 10 mM $CaCl_2$) for SVMP assays at a concentration of 10 mg/mL and 10 dilution steps of a 2-fold serial dilution were prepared. To start the reaction, 50 µL of 2 µM SVSP substrate R110 or 10 µM SVMP substrate ES010 was mixed with 50 µL of each snake venom concentration of the serial dilution. Fluorescence data were recorded using a VICTOR Nivo plate reader at 25 °C. For the SVSP assay, an excitation wavelength of 480 nm and an emission wavelength of 530 nm with 11 kinetic cycles and an interval of 90 sec was used. For the SVMP assay, an excitation wavelength of 320 nm and emission wavelength of 405 nm with 16 kinetic cycles and an interval of 90 sec was used. The reactions were run in duplicate and a blank containing no venom was included.

The rate of relative fluorescence units per second (RFU/s) recorded for each venom concentration was the slope calculated from the linear fitting on its time response curve. The rate values were then plotted against the venom concentration and a non-linear fitting with sigmoidal dose-response equation was used to determine the EC_{50} values (the venom concentration at which half of the maximum RFU/s rate proportional to product conversion rate was observed) using GraphPad Prism 9 software.

Cell viability assay

The N/TERT keratinocyte [24] cell line was cultured in Dulbecco's modified Eagle's medium (DMEM:F12, USA) supplemented with 10% (v/v) fetal bovine serum, 1% (v/v) penicillin-streptomycin and 1 × RMplus supplement [25] at standard conditions (37 °C, 5% CO_2 and 85% humidity). For the cell viability assay, cells were seeded at 4,000 cells/well in 100 µL medium and incubated overnight under standard conditions. Snake venoms were dissolved at a concentration of 10 mg/mL and then 2-fold diluted in 8 dilution steps in sterile PBS. The venom dilutions were then further diluted 1:6 in medium to the maximum concentration of 1 mg/mL and added to each well followed by a 24 h incubation. Thereafter, the CellTiter-Glo luminescent cell viability assay [26] was used to analyze the cytotoxicity of the 26 snake venoms. The assay was performed in triplicates with no venom as negative control (max viability of the cells). IC_{50} values (the venom concentration inducing 50% loss of cell viability) were determined using non-linear fitting with dose response – inhibition equation on the venom dose curves analyzed using GraphPad Prism 9 software.

Thromboelastography (TEG) assay

TEG was run for all viperid venoms (*Bitis arietans*, *B. gabonica*, *B. nasicornis*, *B. rhinoceros*, *Cerastes cerastes*, *Echis leucogaster*, *E. ocellatus*, and *E. pyramidum*) according to a protocol adapted from Seneci *et al* [27] using a TEG 5000 thromboelastogram (Haemonetics). Solutions of 72 µL 25 mM $CaCl_2$, 72 µL 0.25 mM phospholipids (Rossix, catalog no. #PL052), 20 µL Tris-HCl buffer (50 mM Tris + 150 mM NaCl, pH 7.4), and 7 µL crude venom at 1 or 0.1 mg/mL in PBS (final concentration of ~20 µg/mL and ~2 µg/mL, respectively) were mixed in TEG disposable cups (Haemonetics). Lastly, 189 µL of citrated human plasma were added, and the samples were immediately run for at least 30 min. Negative controls were run by replacing venom with 7 µL of PBS. TEG traces (three replicates per

venom concentration per species) were exported as TIFF files and processed in Adobe Photoshop 2022.

Results and discussion

Venom composition

The venom proteomes of the 26 medically most important elapids and vipers from sub-Saharan Africa (Table 2) were determined using a bottom-up proteomics approach, involving the enzymatic digestion of whole venoms, separation and analysis by LC-MS/MS, assignment of the identified proteins to their respective protein families, and calculation of protein family abundances as a percentage of total identified proteins (mol/mol) (Figure 1/Table S1). In addition, the RP-HPLC chromatograms of the venoms are shown in Figure S1.

Elapidae

The elapids included in this study belong to the genera *Naja*, *Hemachatus*, or *Dendroaspis* (Table 2). Of these, the true cobra lineage (*Naja* sp.) is by far the most widespread and diverse group throughout the African continent. To reflect their evolutionary and ecological diversity, African true cobras can be further divided into the three subgenera *Afronaja*, *Boulengerina*, and *Uraeus* [28].

The subgenus *Afronaja* includes all African spitting cobras (*N. ashei*, *N. katiensis*, *N. mossambica*, *N. nigricincta*, *N. nigricollis*, *N. nubiae*, and *N. pallida*), with representative species found from Egypt (*N. nubiae*) to South Africa (*N. mossambica* and *N. nigricincta*) [29]. Despite the widespread distribution and different habitat preferences of *Afronaja* species, their toxin arsenal is remarkably conserved both inter- and intraspecifically [29]. In terms of protein abundance, the proteomic analysis shows that the bulk of their venom consists of 3FTxs (~79%; of which 75% are CTxs) and PLA_2s (~17%), which is in accordance with previous studies

[29,30]. Notably, *N. nubiae* diverges from the general *Afronaja* venom profile and contains a considerable amount of short neurotoxins (sNTxs, ~10%). Envenomings by *N. nubiae* therefore often result in both cytotoxic and neurotoxic clinical manifestations [30] compared to the mostly cytotoxic manifestations seen in envenomings caused by the rest of *Afronaja* species.

The only member of the subgenus *Boulengerina* included in this study was the forest cobra (*N. melanoleuca*) [31], native to the forests and savannahs of central Africa, where it feeds on reptiles, amphibians, birds, small mammals, and even fish [32,33]. Its venom was shown to have a high content of 3FTxs (~64%), of which most were CTxs (~27%), and also a considerable amount of PLA_2s (~28%), which is in agreement with a previous study [19]. Additionally, the venom contained a substantial proportion of long neurotoxins (lNTxs; ~16%), the fourth highest among all snakes in this study, which could explain the neurotoxic manifestations reported after envenoming [34]. One toxin family where the abundance differed from an earlier study was the SVMPs, where Lauridsen *et al* [19] reported an abundance of 9.7%, whereas we only found 0.7%. Plausible explanations for this discrepancy could be a combination of variation between venom batches as a consequence of intraspecific venom variability, differences in the units used to quantify relative protein abundance (mol/mol in this study vs. wt% in Lauridsen et al [19]), and/or the use of different proteomic methods (no decomplexing step prior to mass spectrometry in this study). Notably, the venom profile of *N. melanoleuca* differed from the other two *Naja* subgenera, containing less CTxs than *Afronaja* and more PLA_2s than *Uraeus*.

The *Uraeus* subgenus (*N. anchietae*, *N. annulifera*, *N. haje*, *N. nivea*, and *N. senegalensis*) consists of species with predominantly neurotoxic effects [34]. Like *Afronaja*, this lineage is widespread across the continent from Morocco (*N. haje*) to South Africa (*N. anchietae*, *N. annulifera*, and *N. nivea*) and has a highly diverse diet, which includes amphibians, reptiles, birds and other snakes [35]. The venoms in this subgenus were

predominantly composed of 3FTx (~95%), most of which were CTxs as in the *Afronaja* subgenus, despite the mainly neurotoxic characteristics of *Uraeus* envenomings. This was not the case for *N. haje*, which contained mainly sNTxs (40%) and lNTxs (38%). *N. senegalensis* also contained significant amounts of sNTxs (12%) and lNTxs (23%). Moreover, all *Uraeus* species except *N. anchietae* contained some neurotoxins (at least ~6%), in line with the neurotoxic clinical manifestations associated with envenomings caused by these snakes. For the three snakes where proteomics data was available, our data corroborates the previously reported findings for *N. senegalensis* [36], *N. haje* [37] *and N. nivea* [38]. For *N. annulifera* [39], a previous study reported an abundance 11.18% SVMPs, while our data showed only 0.6%, which, as mentioned previously, could be due to intraspecific venom variability and/or different methods for proteomics and quantification. In contrast to the other *Naja* species, *Uraeus* cobras all showed very low levels of PLA_2s (~0.15%), which were up until recently thought to be almost ubiquitous in snake venoms [40]. Of note, the proteomic venom composition for *N. anchietae* is presented here for the first time, strengthening a previous theory that low levels of PLA_2s are a feature of all snakes within the *Uraeus* subgenus [36].

The rinkhals (*Hemachatus haemachatus*), is a spitting elapid classified in its own monotypic genus despite greatly resembling true cobras in morphology and general biology. This species is native to south-eastern Africa, where it can be found in several ecosystems (*e.g.* savannah, woodland, and shrubland) and is known to prey mainly, although not exclusively, on amphibians [35]. According to our proteomic data, its venom composition is similar to those of the *Afronaja*, spitting cobras, which convergently evolved the ability to spit venom. In fact, *H. haemachatus* venom was shown to have a high content of 3FTxs (~73%) and PLA_2s (~20%). Of the 3FTxs identified, the most abundant were CTxs or CTx-homologs, with a small amount of sNTxs. This correlates with the cytotoxic and neurotoxic clinical manifestations of *H. haemachatus* envenomings [41]. The predominance of 3FTxs and PLA_2s in this species is in

line with a previous study by Sánchez *et al* [8]. However, there are some minor discrepancies, as this previous study detected a larger amount of SVMPs (7 % vs 1.4%).

All four members of the *Dendroaspis* genus (mambas) were included in this study, namely *D. angusticeps*, *D. polylepis*, *D. jamesoni*, and *D. viridis*. Widespread throughout the African continent, these species constitute one of the few predominately arboreal elapid lineages worldwide, cruising through the canopy of rainforest and woodland regions [42,43]. As an exception, *D. polylepis* is often (but not always) more ground-dwelling than its congeners, being commonly found in open savannas and rocky hills [44,45]. Overall, mambas mainly prey on birds and small mammals such as rodents and bats [42,46], although their diet and ecology are poorly known.

Signature components of mamba venoms are the presynaptic neurotoxins called dendrotoxins, which belong to the Kunitz-type protease inhibitor family [12,13]. Dendrotoxins are especially abundant in *D. polylepis* venom, where Kunitz-type protease inhibitors account for 75% of the venom proteins as shown in this and previous studies [18,47]. Conversely, *D. angusticeps* venom mainly consists of 3FTxs, (87%; mainly short-chain aminergic and orphan group toxins), which is once again in accordance with previous findings [42]. The venoms of *D. jamesoni* and *D. viridis* are very similar when comparing the abundance of Kunitz-type protease inhibitors, sNTx, and other 3FTx (Figure 1 / Table S1), but different in terms of lNTx abundance (0.2% and 33%, respectively). Notably, our results are also in agreement with a recent study on venom gland transcriptomics for all four *Dendroaspis* species [17], indicating a general pattern of matching abundance profiles between venom transcriptome and proteome in mambas.

Viperidae

The Viperidae family is represented by the genera *Bitis*, *Cerastes*, and *Echis* in this study. Of these, *Bitis* is the most geographically widespread and taxonomically diverse viperid genus in Africa, with 18 currently recognized species (commonly referred to as African adders) found from Morocco to South Africa [43]. More specifically, *B. rhinoceros* is found in Western Africa from Guinea to Togo, while *B. gabonica* and *B. nasicornis* occurs from Nigeria to Central, Eastern, and Southern Africa [2]. Lastly, *B. arietans* occurs across open woodland, grassland, and semi-arid habitats throughout sub-Saharan Africa, southern Arabia, and Morocco [48]. Large-sized African adders like those included in this study are mostly generalist predators feeding on small mammals, birds, lizards, and occasionally toads [49,50].

The venom compositions of *B. gabonica* and *B. nasicornis* are rather similar and show a high abundance of SVMPs (27% and 24%), SVSPs (17% and 12%), disintegrins (15%), C-type lectins (CTLs)/snaclecs (14% and 32%). SVMPs dominate the venom of *B. arietans* (62%), whereas *B. rhinoceros* venom is particularly rich in PLA_2s (39%) and SVSPs (18%). All these four species have been analyzed previously by Calvete *et al* [51], and overall, our data correlate relatively well with this previous study with some variations observed regarding PLA_2s (*B. gabonica*, *B. nasicornis*, and *B. rhinoceros*), disintegrins (*B. arietans*, *B. gabonica*, and *B. nasicornis*), SVMPs (*B. rhinoceros*), and CTLs (*B. nasicornis*) (Figure 1/Table S1). This discrepancy can be due to several reasons, such as using different methods to generate and analyze the data, as well as intraspecific variation in venom composition [52]. This emphasizes the importance of using the same proteomic approach for cataloguing venom composition of different snakes to enable comparison. Large amounts of rhinocerase 2, a SVSP homolog which contains a H57R mutation [53] was found in the venom of *B. rhinoceros*. Interestingly, this H57R mutation was also found in peptides from *B. gabonica* and *B. nasicornis*, yet in smaller amounts. Such SVSP homologs have previously been detected in *B. gabonica* [54] but this is the first time they have been found in *B. nasicornis*.

The only member of the *Cerastes* genus included in this study is the Saharan horned viper (*Cerastes cerastes*). This species is distributed throughout North Africa and further eastwards as far as Southwestern Israel and Southwestern Saudi Arabia [55]. Like many other viper lineages, *C. cerastes* is an ambush predator, often submerging itself beneath sand to lunge at small rodents and lizards by surprise [56]. Our proteomics analysis of *C. cerastes* venom shows a high abundance of SVSPs (27%) as opposed to a relatively low abundance of CTLs (4%), which is in agreement with other studies [57–59] (Figure 1/Table S1). On the other hand, the largest discrepancy compared to previous reports is observed for SVMPs, which were found to only constitute 8.6% of venom proteins in this study compared to 30-60% reported in literature, and disintegrins, which were found to constitute 43% of venom proteins in this study compared to approximately 10% previously reported in literature [57–59].

The final Viperidae genus included in this study is *Echis*. The family members included are *E. ocellatus*, *E. pyramidum*, and *E. leucogaster*, of which no proteomic data has been reported previously for the latter. *E. ocellatus* and *E. pyramidum* are distributed throughout northern Africa, while *E. leucogaster* occurs in West Africa, isolated areas of the western Sahara, and throughout Algeria [60]. The diet of *Echis* snakes is widely varied, including invertebrates, such as scorpions and centipedes, small mammals, birds, lizards, amphibians, and other snakes [61]. Our proteomics data shows that the venom of *E. pyramidum* and *E. leucogaster* mainly consists of SVMPs with abundances of ~41 and ~42% respectively. In contrast, *E. ocellatus* mainly consists of PLA_2s with an abundance of ~42%, followed by ~26% SVMPs (Figure 1/Table S1). This differs from previous studies, where SVMPs were also reported as the major component of *E. ocellatus* venom with abundances of ~70% [62,63]. Again, different methods used to analyze the venom composition and the origin of the snakes milked to obtain the venoms may be underlying reasons for the observed differences. SVSPs of *Echis* venom comprise less than 2% of the whole venom (Figure 1/Table S1), which is in

accordance with previous reports [62,63]. In agreement with a previous study showing that the genetic variability between *E. pyramidum* and *E. leucogaster* is very low [64], our proteomics data show that the venom composition of *E. leucogaster* is quite similar to that of *E. pyramidum*. Strikingly, the amount of disintegrins was found to be 11% for *E. ocellatus* and less than 0.01% for *E. pyramidum* and *E. leucogaster* (Figure 1/Table S1). Extensive, likely diet-driven, interspecific venom variation has been documented in *Echis* representatives at the transcriptome and proteome level [62], and in functional toxicity studies [65–67]. It is plausible that this interspecific variation can explain the differences between our results and previous analyses of *E. pyramidum* and *E. ocellatus* venoms.

In vitro **functional characterization of whole venoms**

To evaluate and compare functional activities of sub-Saharan Africa's 26 medically most relevant elapid and viper venoms, we determined the concentrations of snake venom resulting in 50% product conversion at a fixed substrate concentration (EC_{50} values) in PLA_2, SVSP, and SVMP enzymatic activity assays, and the concentrations of snake venom reducing the cell viability by 50% (IC_{50} values) in a cell viability assay. Lower EC_{50} or IC_{50} values indicate more potent activity of the analyzed toxins in the whole venom.

Family	Genus (sub-genus)	Snake	Cell viability assay IC_{50} (µg/mL)	Enzymatic activity assays EC_{50} (µg/mL) PLA$_2$	SVSP	SVMP
Elapidae	*Dendroaspis*	*D. angusticeps*	ND	ND	-	ND
		D. jamesoni	ND	ND	-	241.3 ± 31.1
		D. polylepis	ND	ND	-	ND
		D. viridis	ND	ND	-	135.9 ± 32.2
	Hemachatus	*H. haemachatus*	27.4 ± 2.0	93.7 ± 0.3	-	ND
	Naja (Afronaja)	*N. ashei*	12.1 ± 0.1	38.0 ± 0.1	-	666.6 ± 223.5
		N. katiensis	21.3 ± 0.7	37.1 ± 0.1	-	949.2 ± 395.2
		N. mossambica	18.7 ± 1.3	25.4 ± 0.1	-	428.3 ± 79.7
		N. nigricincta	7.2 ± 0.1	15.5 ± 0.4	-	ND
		N. nigricollis	20.8 ± 1.1	33.5 ± 0.1	-	ND
		N. nubiae	13.6 ± 0.3	20.3 ± 0.1	-	857.5 ± 232.1
		N. pallida	17.5 ± 0.7	17.7 ± 0.5	-	ND
	Naja (Boulengerina)	*N. melanoleuca*	6.9 ± 0.1	80.7 ± 0.4	-	421.3 ± 68.4
	Naja (Uraeus)	*N. anchietae*	15.7 ± 1.4	301.7 ± 0.6	-	ND
		N. annulifera	21.1 ± 1.5	354.8 ± 0.5	-	76.9 ± 9.5
		N. haje	9.9 ± 0.4	249.7 ± 0.6	-	769.6 ± 231.0
		N. nivea	14.5 ± 0.2	705.4 ± 38.8	-	375.6 ± 146.4
		N. senegalensis	4.7 ± 0.2	366.6 ± 10.1	-	ND
Viperidae	*Bitis*	*B. arietans*	5.7 ± 0.3	ND	523.9 ± 45.7	3.3 ± 0.7
		B. gabonica	3.7 ± 0.2	37.0 ± 1.0	104.2 ± 18.0	38.1 ± 7.0
		B. nasicornis	5.5 ± 0.2	35.0 ± 1.0	161.5 ± 20.7	53.9 ± 7.8
		B. rhinoceros	2.8 ± 0.1	ND	1150.0 ± 242.8	69.2 ± 11.7
	Cerastes	*C. cerastes*	2.0 ± 0.1	144.0 ± 7.0	24.8 ± 3.7	57.6 ± 7.1
	Echis	*E. leucogaster*	3.9 ± 0.2	357.0 ± 17.0	ND	13.5 ± 3.7
		E. ocellatus	2.1 ± 0.1	857.0 ± 90.0	ND	2.1 ± 0.4
		E. pyramidum	6.5 ± 0.1	435.0 ± 13.0	ND	9.2 ± 1.2

High activity
Low activity
Not determined (ND)
Not tested

Table 2: Functional *in vitro* activities of whole venoms from the 26 medically most relevant elapids and vipers in sub-Saharan Africa. Colour scales indicate the IC_{50} and EC_{50} values. ND: not determined (activity too low), -: not tested.

PLA_2 enzymatic activity

Secreted PLA_2s are one of the major components of many animal venoms. These 13–15 kDa enzymes need Ca^{2+} ions to catalyze the hydrolysis of phospholipids [68]. However, it is noteworthy that some PLA_2s have lost their enzymatic activity during evolution [69]. In the non-catalytic PLA_2s, the catalytic residue D49 is mutated to another amino acid (*e.g.* lysine, serine, asparagine, glutamine, or arginine), resulting in a conformational change of the Ca^{2+}

binding loop that prevents the reaction by hindering Ca^{2+} coordination, which is essential for catalysis [70,71]. Despite sharing 40–99% amino acid sequence identity and highly conserved three dimensional structures, snake venom PLA_2s display a wide variety of pharmacological activities, including neurotoxic, myotoxic, cytotoxic, anticoagulant, and hemolytic effects [72].

Among the 18 snake species of the Elapidae family included in this study, the subgenus *Afronaja* shows the highest PLA_2 activity with EC_{50} values of 15 - 38 µg/mL (Figure 2A, Table 2). The subgenus *Boulengerina* and genus *Hemachatus* have moderate PLA_2 activity with EC_{50} values of 80 - 90 µg/mL, while the subgenus *Uraeus* exhibits low PLA_2 activity with EC_{50} values above 200 µg/mL. Finally, *Dendroaspis* venoms display the weakest PLA_2 activity (EC_{50} values over 1 mg/mL), which is in agreement with previous findings [18,73]. The PLA_2 activity of elapid snake venoms can therefore be ranked in the following order: *Afronaja* > *Boulengerina* > *Hemachatus* > *Uraeus* > *Dendroaspis*. This is in agreement with a previous publication on PLA_2 activity of the three *Naja* subgenera [40] and correlates to the relative abundance of PLA_2s in our proteomics data, except for *N. melanoleuca* which exhibits the highest PLA_2 abundance amongst the elapids but slightly lower activity than the 7 snakes from the *Afronaja* subgenus (Figure 2A, Table 2).

Within the 8 snakes from the Viperidae family, *B. gabonica* and *B. nasicornis* show the lowest EC_{50} values for PLA_2 activity (~40 µg/mL; Figure 2B, Table 2), which is comparable to that of the subgenus *Afronaja* from the Elapidae family. The high PLA_2 activity of *B. gabonica* and *B. nasicornis* venom is in agreement with a previous publication [51], but differs to our proteomics data, which shows a low PLA_2 abundance (Figure 2C). Notably, two other species from the *Bitis* genus, *i.e.*, *B. arietans* and *B. rhinoceros,* showed weak PLA_2 activity, likely due to high abundances of mutated PLA_2s in their venoms (Figure 2C). Similarly, the three species from the *Echis* genus exhibited high relative abundances of PLA_2, but weak enzymatic activity, which can be explained by high amounts of non-catalytic PLA_2s found in

Echis venoms (Figure 2C). This result is consistent with a previous study reporting high myotoxic activity, but low enzymatic activity, of S49 PLA_2s in *E. ocellatus* and *E. pyramidum* venoms [74]. The *Cerastes* genus displayed the second highest PLA_2 activity, with an EC_{50} value of 144 µg/mL. In general, our data demonstrate that there is a high correlation between PLA_2 activity and PLA_2 relative abundance for most of the viperid snake venoms.

SVSP enzymatic activity

SVSPs are a member of the S1 peptidase family, which catalyzes the cleavage of covalent peptide bonds of proteins via the conserved catalytic triad H57-D102-S195, in which serine serves as the nucleophilic amino acid at the active site [75]. These 26–67 kDa enzymes affect the coagulation cascade, the fibrinolytic and kallikrein-kinin systems, and cause hemostatic imbalances in victims [76]. In this relation, SVSPs can be classified as either procoagulant, anticoagulant, platelet-aggregating, or activators of fibrinolysis [77].

Our proteomic data showed negligible amounts of SVSPs in the elapid venoms (Figure 3B) and, therefore, EC_{50} values of SVSPs were only determined for viperid venoms (Figure 3A, Table 2). *C. cerastes* showed the lowest EC_{50} value (25 µg/mL), which correlates with the high SVSP abundance in its venom (27.5%, Figure 3B). Within the genus *Bitis*, the SVSP activity of *B. gabonica* and *B. nasicornis* is moderate, with EC_{50} values of ~100 µg/mL, whereas *B. arietans* and *B. rhinoceros* demonstrate weak SVSP activity with EC_{50} values between 500 - 1000 µg/mL (Figure 3A, Table 2). Although *B. rhinoceros* exhibits the second highest SVSP abundance amongst the 8 vipers included in this study, its EC_{50} value is in the high range. This could be because, as mentioned before, that an SVSP homolog with a catalytic site mutation [53] was found in our proteomic analysis. Finally, the genus *Echis* showed the weakest SVSP activity with EC_{50} values above 1 mg/mL, which agrees with their low SVSP abundance (below 2%, Figure 3B).

SVMP enzymatic activity

The Zn^{2+} dependent SVMPs are one of the most abundant toxins in viperid venoms [7] mainly responsible for inducing systemic hemorrhage after envenomings with these snakes. There are three major classes of SVMP: P-I contains only a metalloproteinase (M) domain, P-II contains an M domain and a disintegrin (D) domain, and the most complex P-III class is composed of an M domain, a D domain and a cysteine-rich (C) domain.

All elapids show high EC_{50} values of at least 100 μg/mL, except *N. annulifera* with a value of 80 μg/mL. This is in agreement with previously published data showing that SVMP is the second most abundant protein family in *N. annulifera* venom after 3FTxs [39]. SVMPs in the *Dendroaspis* species have low abundance but a high activity in *D. jamesoni* and *D. viridis* (EC_{50} values of 241 and 136 μg/mL respectively). This is in agreement with an earlier study where SVMP dependent anticoagulant activity was observed in *Dendroaspis* species despite the low SVMP abundance [78]. Among the 8 viperids included in this study, *B. arietans* shows the lowest EC_{50} value (3.3 μg/mL), while the 3 venoms from the *Echis* subgenus show high amounts of SVMPs (Figure 4C) with EC_{50} values between ~2–14 μg/mL (Figure 4B, Table 2). *B. arietans* has an EC_{50} value in the same range as the *Echis* venom, whereas all other *Bitis* species have EC_{50} values around 40–50 μg/mL.

Cell viability

CTxs and PLA_2s are known to, either individually or synergistically, interfere with and disrupt the integrity of cellular membranes, leading to irreversible damage and cell death [25,79]. In snake venoms, cytotoxins are mainly found in the genera *Naja* and *Hemachatus* of the Elapidae family [8], while PLA_2s are found in all venomous snake families, including Elapidae and Viperidae [80]. Therefore, the cytotoxicity of all elapid and viperid venoms

included in this study was evaluated using an immortalized human keratinocyte cell line, which has been reported to be sensitive to snake venom cytotoxins and PLA_2s [25].

Treatment of the cells with venoms resulted in a concentration-dependent inhibition of cell viability (Figure 5). As expected, the vipers venoms were more potent (IC_{50} 2.0–6.5 µg/mL) than venoms from the elapids (IC_{50} 4.7 to > 100 µg/mL). Amongst the Elapidae, four species from the *Naja* genus, *i.e.*, *N. senegalensis*, *N. melanoleuca*, *N. nigricincta*, and *N. haje*, showed IC_{50} values close to those of the Viperidae (below 10 µg/mL), while the *Dendroaspis* genus demonstrated IC_{50} values above 100 µg/mL. These results are in alignment with a high abundance of cytotoxins in the *Naja* genus and PLA_2s in the Viperidae family, and a lack of these two toxin families in the *Dendroaspis* genus.

Thromboelastography

The blood coagulation cascade is a primary target for many snake venom toxins due to its pivotal role in maintaining homeostasis, and most major venomous snake families possess toxins in their venoms that can interfere with this system. This is particularly evident in (although not exclusive to) vipers, whose venoms are generally dominated by proteins that cause coagulopathies (*e.g.*, SVMPs, SVSPs, and disintegrins) [81,82]. Thus, we assessed the coagulotoxic effects of the venoms of all viper species included in this study via thromboelastography (TEG) by incubating whole venom with human plasma and physiological cofactors of coagulation (*i.e.*, calcium and phospholipids).

All venoms were tested, and presented similar activity, at ~2 µg/mL and ~20 µg/mL except *B. arietans*, which showed inconclusive results at ~2 µg/mL. Both anti- and procoagulant effects were observed on a broadly genus-specific basis. More specifically, venoms from all *Bitis* species, except *B. nasicornis*, displayed an overall strong anticoagulant activity, with no visible clot formation (Figure 6).

Unlike *Bitis*, *C. cerastes* venom produced a detectable, stable clot almost immediately after assay initiation (Figure 6). Interestingly, other researchers have reported that the venom of this species exerts both pro- and anticoagulant effects in a concentration-dependent manner, whereby low venom concentrations (≤ 200 µg/mL), as used in our study, enhanced blood clotting in agreement with our observations, while higher amounts disrupted coagulation [83].

Lastly, an even stronger procoagulant activity than seen for *C. cerastes* was observed for the three *Echis* representatives included in this study (Figure 6). This is not surprising, as a signature trait of most *Echis* species is the presence of exceptionally potent prothrombin activators (all part of the SVMP family) in their venoms, which results in uncontrolled formation of fibrin clots due to excessive production of thrombin [84–87]. This agrees with our proteomic data where *Echis* venoms show high amounts of SVMPs for all three species.

Conclusion

In this study, we systematically analyzed and compared the proteomics and *in vitro* functional activity of multiple snake venoms and provide toxicovenomic profiles of 26 of sub-Saharan Africa's medically most important elapids and vipers. To the best of our knowledge, the venom composition of *N. anchietae* and *E. leucogaster* are presented here for the first time. Overall, our data show that the elapid venoms contained large amounts of neurotoxic and cytotoxic 3FTxs and PLA_2s, whereas the viper venoms were dominated by hemotoxic and/or cytotoxic PLA_2s, SVMPs, and SVSPs, as expected based on clinical manifestations observed for elapid and viperid envenoming [7].

The high-throughput, label-free, quantitative proteomics approach presented here comes with some limitations. During mass spectrometry, proteins are identified through mapping of the peptide sequence to a database. The lack of a comprehensive database may thus

result in false negatives; if the sequence of a protein is not present in the database, it cannot be detected. It is also important to keep in mind that peptides from highly similar isoforms may be difficult to map back to their parent proteins, resulting in false positives (*e.g.,* detection of a disintegrin instead of a SVMP).

In respect to functional activity, the subgenus *Afronaja*, together with *B. gabonica,* and *B. nasicornis* showed the highest enzymatic PLA_2 activity, which highlights the importance of catalytic PLA_2s in relation to the clinical manifestations observed after envenoming with these snakes, such as hemolytic and anticoagulant effects [88,89]. When comparing the enzymatic SVSP activity among the vipers, the abundance of active SVSPs in the venoms showed a good correlation with activity; for the outlier *B. rhinoceros*, the high abundance, but low activity of SVSPs, can be explained by the high proportion of catalytically inactive SVSP homologs in its venom. All viper venoms were shown to possess high SVMP activity with low EC_{50} values, 10-100 times higher activity than most of the elapid venoms, where only *N. annulifera* venom showed activity in the same range as the vipers. Given that *N. annulifera* has been shown in a previous study to possess a substantial amount of SVMP in its venom [39], higher than many other cobra species, this is not too surprising.

The coagulotoxic effect of the viper venoms included in this study was assessed by TEG, showing a procoagulant effect for venoms from *C. cerastes* and the genus *Echis*. In contrast, all *Bitis* species showed strong anticoagulant activity except *B. nasicornis*, which also showed an anticoagulant activity but to a lesser extent. Finally, cell viability of a keratinocyte cell line was inhibited by addition of snake venoms from all snake species, except the ones from the genus of *Dendroaspis.* This is not surprising, given that *Dendroaspis* venoms are known to be highly neurotoxic, cause very little tissue damage, and display very low enzymatic activities [18,73].

Some inconsistencies were observed between the relative abundance of certain toxin families and whole venom activity in their respective *in vitro* functional assays. For example, our data show that the venom of *N. annulifera, D. viridis,* and *D. jamesoni* had high SVMP activity despite low abundance of such proteins. When it comes to *in vitro* assays for characterizing toxin functions, a limitation of the present study is the lack of assays to assess the activity of neurotoxins, as several species included herein (*e.g.,* mambas and most *Uraeus* cobras) possess predominately neurotoxic venoms [18,34,73].

Overall, this study can provide a foundation for further studies of snake biology and evolution, for which we recommend an integrated approach combining genomics, transcriptomics, and proteomics to provide information on gene expression and other molecular mechanisms linked to phenotypic diversity [1]. Moreover, the toxicovenomic profiles elucidated in this study may aid in the development of effective antivenoms through better understanding of the behavior of snake venoms and their roles as drug targets.

List of abbreviations

NOB	4-nitro-3-(octanoyloxy)benzoic acid
nAChRs	Acetylcholine receptors
CTLs	C-type lectins
CTxs	Cytotoxins
LC	Liquid chromatography
lNTxs	Long neurotoxins
PLA_2s	Phospholipase A_2s
FRU/s	Relative fluorescence units per second
RP-HPLC	Reversed-phase high-performance liquid chromatography
sNTxs	Short neurotoxins

SVMPs	Snake venom metalloproteinases
SVSPs	Snake venom serine proteinases
3FTxs	Three-finger toxins
TEG	Thromboelastography
TFA	Trifluoroacetic acid

Data accessibility

The data sets supporting the results of this article are available in the GigaScience GigaDB repository. Results from the *in vitro* functional activity assays are available at [DOI pending]. Results from the proteomics characterizations are available at [DOI pending].

Additional files

Supplementary Figure S1: RP-HPLC chromatograms of the whole venoms of 26 sub-Saharan snakes.

Supplementary Table S1: Composition of the whole venoms of 26 sub-Saharan snakes.

Competing interest

All authors declare no conflict of interest.

Funding

This research was founded by a grant from Wellcome [221702/z/20/z]

Authors' contribution

G.T.T.N, C.O.B, A.H.L, and A.L conceived the study. G.T.T.N, C.O.B, Y.W, L.S, A.G.C, and I.C.P performed laboratory experiments and analyzed the data. A.H.L and A.L supervised the

study. G.T.T.N, C.O.B, Y.W, L.S., A.G.C, S.A, A.H.L, and A.L drafted the manuscript. All authors read and approved the final manuscript.

Acknowledgments:

We thank Lars Winther and Peter Ådal Nielsen (Tissue-Link™) for granting us use of the TEG®5000 thromboelastogram and supplying us with reagents. We also thank Dr. Christina N. Zdenek (School of Biological Sciences, the University of Queensland, Australia) for valuable advice in devising the TEG protocol. Mass spectrometry analysis was performed at the DTU Proteomics Core, Technical University of Denmark.

Figure legends:

Figure 1. Composition of the whole venoms of the 26 medically most important elapids and vipers from sub-Saharan Africa. Toxins are grouped according to protein families and expressed as a percentage of total identified proteins (mol/mol). CTx: cytotoxin, sNTx: short neurotoxin, lNTx: long neurotoxin, 3FTx: three-finger toxin, CTL: C-type lectin, PLA_2: phospholipase A_2, SVMP: snake venom metalloproteinase, SVSP: snake venom serine proteinase.

Figure 2. PLA_2 enzymatic activity of whole venoms of elapids (A) and vipers (B) at different venom concentrations. Absorbances at 405 nm were normalized by subtracting values of the negative control (absence of venom). Error bars: SD from two independent measurements. (C) Relative abundance of enzymatically active PLA_2s (D49) and enzymatically inactive PLA_2s (mut) in 26 sub-Saharan snake venoms. A heatmap displaying EC_{50} values for each snake venom is plotted above the corresponding abundance.

Figure 3. (A) SVSP enzymatic activity of viper whole venoms at different venom concentrations. RFU: relative fluorescence unit. Error bars: SD from two independent measurements. (B) Relative abundance of SVSPs in 26 sub-Saharan snake venoms. A heatmap displaying EC_{50} values for each snake venom is plotted above the corresponding abundance.

Figure 4. SVMP enzymatic activity of the whole venoms of elapids (A) and vipers (B) at different venom concentrations. RFU: relative fluorescence unit. Error bars: SD from two independent measurements. (C) Relative abundance of SVMP sub-families in 26 sub-Saharan snake venoms. A heatmap displaying EC_{50} values for each snake venom is plotted above the corresponding abundance.

Figure 5. Cell viability of the N/TERT keratinocyte cell line after addition of different concentrations of whole venoms of elapids (A) and vipers (B). The negative control value (without venom) was set to 100%. Error bars: SD from two independent measurements.

Figure 6. Overlaid thromboelastography traces showing the ability of the different venoms (2 µg/mL for all species except *B. arietans*, 20 µg/mL) to clot plasma relative to a spontaneous control in one hour. Time is plotted horizontally and amplitude (clotting strength) is plotted vertically. Eight representatives of the Viperidae family, which possess procoagulant (*C. cerastes*, *E. leucogaster*, *E. ocellatus*, and *E. pyramidum*), and anticoagulant (*B. arietans*, *B. gabonica*, *B. nasicornis*, and *B. rhinoceros*) venom are depicted. Blue traces represent spontaneous clot controls and red traces represent samples (n = 3).

References

1. Rao W, Kalogeropoulos K, Allentoft ME, Gopalakrishnan S, Zhao W, Workman CT, et al.. The rise of genomics in snake venom research: recent advances and future perspectives. *GigaScience*. 2022; doi: 10.1093/gigascience/giac024.

2. Spawls S, Branch B. The Dangerous Snakes of Africa. Bloomsbury Publishing;

3. O'Shea M. Venomous Snakes of the World. Princeton University Press;

4. Kasturiratne A, Wickremasinghe AR, de Silva N, Gunawardena NK, Pathmeswaran A, Premaratna R, et al.. The Global Burden of Snakebite: A Literature Analysis and Modelling Based on Regional Estimates of Envenoming and Deaths. Winkel K, editor. *PLoS Med*. 2008; doi: 10.1371/journal.pmed.0050218.

5. Babo Martins S, Bolon I, Alcoba G, Ochoa C, Torgerson P, Sharma SK, et al.. Assessment of the effect of snakebite on health and socioeconomic factors using a One Health perspective in the Terai region of Nepal: a cross-sectional study. *The Lancet Global Health*. 2022; doi: 10.1016/S2214-109X(21)00549-0.

6. Harrison RA, Hargreaves A, Wagstaff SC, Faragher B, Lalloo DG. Snake Envenoming: A Disease of Poverty. White J, editor. *PLoS Negl Trop Dis*. 2009; doi: 10.1371/journal.pntd.0000569.

7. Gutiérrez JM, Calvete JJ, Habib AG, Harrison RA, Williams DJ, Warrell DA. Snakebite envenoming. *Nat Rev Dis Primers*. 2017; doi: 10.1038/nrdp.2017.63.

8. Sánchez A, Segura Á, Pla D, Munuera J, Villalta M, Quesada-Bernat S, et al.. Comparative venomics and preclinical efficacy evaluation of a monospecific Hemachatus antivenom towards sub-Saharan Africa cobra venoms. *Journal of Proteomics*. 2021; doi: 10.1016/j.jprot.2021.104196.

9. Adukauskienė D, Varanauskienė E, Adukauskaitė A. Venomous Snakebites. *Medicina*. 2011; doi: 10.3390/medicina47080061.

10. Méndez I, Gutiérrez JM, Angulo Y, Calvete JJ, Lomonte B. Comparative study of the cytolytic activity of snake venoms from African spitting cobras (Naja spp., Elapidae) and its neutralization by a polyspecific antivenom. *Toxicon*. 2011; doi: 10.1016/j.toxicon.2011.08.018.

11. Nirthanan S, Gwee MCE. Three-Finger α-Neurotoxins and the Nicotinic Acetylcholine Receptor, Forty Years On. *J Pharmacol Sci*. 2004; doi: 10.1254/jphs.94.1.

12. Harvey AL. Twenty years of dendrotoxins. *Toxicon*. 2001; doi: 10.1016/S0041-0101(00)00162-8.

13. Harvey AL, Anderson AJ. Dendrotoxins: Snake toxins that block potassium channels and facilitate neurotransmitter release. *Pharmacology & Therapeutics*. 1985; doi: 10.1016/0163-7258(85)90036-1.

14. Gutiérrez J, Escalante T, Rucavado A, Herrera C, Fox J. A Comprehensive View of the Structural and Functional Alterations of Extracellular Matrix by Snake Venom Metalloproteinases (SVMPs): Novel Perspectives on the Pathophysiology of Envenoming. *Toxins*. 2016; doi: 10.3390/toxins8100304.

15. Gutiérrez J. Snake venom metalloproteinases:Their role in the pathogenesis of local tissue damage. *Biochimie*. 2000; doi: 10.1016/S0300-9084(00)01163-9.

16. Serrano SMT. The long road of research on snake venom serine proteinases. *Toxicon*. 2013; doi: 10.1016/j.toxicon.2012.09.003.

17. Ainsworth S, Petras D, Engmark M, Süssmuth RD, Whiteley G, Albulescu L-O, et al.. The medical threat of mamba envenoming in sub-Saharan Africa revealed by genus-wide analysis of venom composition, toxicity and antivenomics profiling of available antivenoms. *Journal of Proteomics*. 2018; doi: 10.1016/j.jprot.2017.08.016.

18. Laustsen AH, Lomonte B, Lohse B, Fernández J, Gutiérrez JM. Unveiling the nature of black mamba (Dendroaspis polylepis) venom through venomics and antivenom immunoprofiling: Identification of key toxin targets for antivenom development. *Journal of Proteomics*. 2015; doi: 10.1016/j.jprot.2015.02.002.

19. Lauridsen LP, Laustsen AH, Lomonte B, Gutiérrez JM. Exploring the venom of the forest cobra snake: Toxicovenomics and antivenom profiling of Naja melanoleuca. *Journal of Proteomics*. 2017; doi: 10.1016/j.jprot.2016.08.024.

20. Damm M, Hempel B-F, Süssmuth RD. Old World Vipers—A Review about Snake Venom Proteomics of Viperinae and Their Variations. *Toxins*. 2021; doi: 10.3390/toxins13060427.

21. Wagstaff SC, Sanz L, Juárez P, Harrison RA, Calvete JJ. Combined snake venomics and venom gland transcriptomic analysis of the ocellated carpet viper, Echis ocellatus. *Journal of Proteomics*. 2009; doi: 10.1016/j.jprot.2008.10.003.

22. Calvete JJ. Proteomic tools against the neglected pathology of snake bite envenoming. *Expert Review of Proteomics*. 2011; doi: 10.1586/epr.11.61.

23. Holzer M, Mackessy SP. An aqueous endpoint assay of snake venom phospholipase A2. *Toxicon*. 1996; doi: 10.1016/0041-0101(96)00057-8.

24. Dickson MA, Hahn WC, Ino Y, Ronfard V, Wu JY, Weinberg RA, et al.. Human Keratinocytes That Express hTERT and Also Bypass a p16 ^{INK4a} -Enforced Mechanism That Limits Life Span Become Immortal yet Retain Normal Growth and Differentiation Characteristics. *Mol Cell Biol*. 2000; doi: 10.1128/MCB.20.4.1436-1447.2000.

25. Pucca MB, Ahmadi S, Cerni FA, Ledsgaard L, Sørensen CV, McGeoghan FTS, et al.. Unity Makes Strength: Exploring Intraspecies and Interspecies Toxin Synergism between Phospholipases A2 and Cytotoxins. *Front Pharmacol*. 2020; doi: 10.3389/fphar.2020.00611.

26. Riss TL, Moravec RA, Niles AL. Cytotoxicity Testing: Measuring Viable Cells, Dead Cells, and Detecting Mechanism of Cell Death. In: Stoddart MJ, editor. *Mammalian Cell Viability*. Totowa, NJ: Humana Press;

27. Seneci L, Zdenek CN, Chowdhury A, Rodrigues CFB, Neri-Castro E, Bénard-Valle M, et al.. A Clot Twist: Extreme Variation in Coagulotoxicity Mechanisms in Mexican Neotropical Rattlesnake Venoms. *Front Immunol*. 2021; doi: 10.3389/fimmu.2021.612846.

28. Wallach V, Wüster W, Broadley DG. In praise of subgenera: taxonomic status of cobras of the genus Naja Laurenti (Serpentes: Elapidae). *Zootaxa*. 2009; doi: 10.11646/zootaxa.2236.1.2.

29. Hus K, Buczkowicz J, Petrilla V, Petrillová M, Łyskowski A, Legáth J, et al.. First Look at the Venom of Naja ashei. *Molecules*. 2018; doi: 10.3390/molecules23030609.

30. Petras D, Sanz L, Segura Á, Herrera M, Villalta M, Solano D, et al.. Snake Venomics of African Spitting Cobras: Toxin Composition and Assessment of Congeneric Cross-Reactivity of the Pan-African EchiTAb-Plus-ICP Antivenom by Antivenomics and Neutralization Approaches. *J Proteome Res*. 2011; doi: 10.1021/pr101040f.

31. Wüster W, Chirio L, Trape J-F, Ineich I, Jackson K, Greenbaum E, et al.. Integration of nuclear and mitochondrial gene sequences and morphology reveals unexpected diversity in the forest cobra (Naja melanoleuca) species complex in Central and West Africa (Serpentes: Elapidae). *Zootaxa*. 2018; doi: 10.11646/zootaxa.4455.1.3.

32. Luiselli L, Angelici FM, Akani GC. Comparative feeding strategies and dietary plasticity of the sympatric cobras Naja melanoleuca and Naja nigricollis in three diverging Afrotropical habitats. 80:92002;

33. Luiselli L, Akani GC, Corti C, Angelici FM. Is sexual size dimorphism in relative head size correlated with intersexual dietary divergence in West African forest cobras, Naja melanoleuca? *CTOZ*. 2002; doi: 10.1163/18759866-07104004.

34. Guidelines for the prevention and clinical management of snakebite in Africa. Brazzaville: World Health Organization;

35. Shine R, Branch WR, Webb JK, Harlow PS, Shine T, Keogh JS. Ecology of cobras from southern Africa. *Journal of Zoology*. 2007; doi: 10.1111/j.1469-7998.2006.00252.x.

36. Wong KY, Tan KY, Tan NH, Tan CH. A Neurotoxic Snake Venom without Phospholipase A2: Proteomics and Cross-Neutralization of the Venom from Senegalese Cobra, Naja senegalensis (Subgenus: Uraeus). *Toxins*. 2021; doi: 10.3390/toxins13010060.

37. Malih I, Ahmad rusmili MR, Tee TY, Saile R, Ghalim N, Othman I. Proteomic analysis of Moroccan cobra Naja haje legionis venom using tandem mass spectrometry. *Journal of Proteomics*. 2014; doi: 10.1016/j.jprot.2013.11.012.

38. Kazandjian TD, Petras D, Robinson SD, van Thiel J, Greene HW, Arbuckle K, et al.. Convergent evolution of pain-inducing defensive venom components in spitting cobras. *Science*. American Association for the Advancement of Science; 2021; doi: 10.1126/science.abb9303.

39. Tan KY, Wong KY, Tan NH, Tan CH. Quantitative proteomics of Naja annulifera (sub-Saharan snouted cobra) venom and neutralization activities of two antivenoms in Africa. *International Journal of Biological Macromolecules*. 2020; doi: 10.1016/j.ijbiomac.2020.04.173.

40. Tan C, Wong K, Tan N, Ng T, Tan K. Distinctive Distribution of Secretory Phospholipases A2 in the Venoms of Afro-Asian Cobras (Subgenus: Naja, Afronaja, Boulengerina and Uraeus). *Toxins*. 2019; doi: 10.3390/toxins11020116.

41. Blaylock R. The identification and syndromic management of snakebite in South Africa. *South African Family Practice*. 2005; doi: 10.1080/20786204.2005.10873288.

42. Luiselli L, Angelici FM, Akani GC. Large elapids and arboreality: the ecology of Jameson's green mamba (Dendroaspis jamesoni) in an Afrotropical forested region. *CTOZ*. 2000; doi: 10.1163/18759866-06903001.

43. Shine R, Spawls S. An ecological analysis of snakes captured by C.J.P. Ionides in eastern Africa in the mid-1900s. *Sci Rep*. 2020; doi: 10.1038/s41598-020-61974-4.

44. Hakansson T, Madsen T. On the Distribution of the Black Mamba (Dendroaspis polylepis) in West Africa. *Journal of Herpetology*. 1983; doi: 10.2307/1563464.

45. Marais J. A complete guide to the snakes of southern Africa. 1st ed. [Cape Town: Struik]

46. Maritz B, Barends JM, Mohamed R, Maritz RA, Alexander GJ. Repeated dietary shifts in elapid snakes (Squamata: Elapidae) revealed by ancestral state reconstruction. *Biological Journal of the Linnean Society*. 2021; doi: 10.1093/biolinnean/blab115.

47. Petras D, Heiss P, Harrison RA, Süssmuth RD, Calvete JJ. Top-down venomics of the East African green mamba, Dendroaspis angusticeps , and the black mamba, Dendroaspis polylepis, highlight the complexity of their toxin arsenals. *Journal of Proteomics*. 2016; doi: 10.1016/j.jprot.2016.06.018.

48. Barlow A, Wüster W, Kelly CMR, Branch WR, Phelps T, Tolley KA. Ancient habitat shifts and organismal diversification are decoupled in the African viper genus *Bitis* (Serpentes: Viperidae). *J Biogeogr*. 2019; doi: 10.1111/jbi.13578.

49. Luiselli L, Akani GC. Diet of sympatric Gaboon Vipers (*Bitis gabonica*) and Nose-horned Vipers (*Bitis nasicornis*) in southern Nigeria. *African Journal of Herpetology*. 2003; doi: 10.1080/21564574.2003.9635485.

50. Glaudas X, Kearney TC, Alexander GJ. Museum Specimens Bias Measures of Snake Diet: A Case Study Using the Ambush-Foraging Puff Adder (*Bitis arietans*). *Herpetologica*. 2017; doi: 10.1655/HERPETOLOGICA-D-16-00055.

51. Calvete JJ, Escolano J, Sanz L. Snake Venomics of Bitis Species Reveals Large Intragenus Venom Toxin Composition Variation: Application to Taxonomy of Congeneric Taxa. *J Proteome Res*. American Chemical Society; 2007; doi: 10.1021/pr0701714.

52. Chippaux J-P, Williams V, White J. Snake venom variability: methods of study, results and interpretation. *Toxicon*. 1991; doi: 10.1016/0041-0101(91)90116-9.

53. Vaiyapuri S, Wagstaff SC, Harrison RA, Gibbins JM, Hutchinson EG. Evolutionary Analysis of Novel Serine Proteases in the Venom Gland Transcriptome of Bitis gabonica rhinoceros. Ho PL, editor. *PLoS ONE*. 2011; doi: 10.1371/journal.pone.0021532.

54. Francischetti IMB, My-Pham V, Harrison J, Garfield MK, Ribeiro JMC. Bitis gabonica (Gaboon viper) snake venom gland: toward a catalog for the full-length transcripts (cDNA) and proteins. *Gene*. 2004; doi: 10.1016/j.gene.2004.03.024.

55. Schneemann M, Cathomas R, Laidlaw ST, El Nahas AM, Theakston RDG, Warrell DA. Life-threatening envenoming by the Saharan horned viper (Cerastes cerastes) causing micro-angiopathic haemolysis, coagulopathy and acute renal failure: clinical cases and review. *QJM*. 2004; doi: 10.1093/qjmed/hch118.

56. Young BA, Morain M. Prey localization in the Saharan sand vipers. :5.

57. Bazaa A, Marrakchi N, El Ayeb M, Sanz L, Calvete JJ. Snake venomics: Comparative analysis of the venom proteomes of the Tunisian snakesCerastes cerastes, Cerastes vipera andMacrovipera lebetina. *Proteomics*. 2005; doi: 10.1002/pmic.200402024.

58. Fahmi L, Makran B, Pla D, Sanz L, Oukkache N, Lkhider M, et al.. Venomics and antivenomics profiles of North African Cerastes cerastes and C. vipera populations reveals a potentially important therapeutic weakness. *Journal of Proteomics*. 2012; doi: 10.1016/j.jprot.2012.02.021.

59. Ozverel CS, Damm M, Hempel B-F, Göçmen B, Sroka R, Süssmuth RD, et al.. Investigating the cytotoxic effects of the venom proteome of two species of the Viperidae family (Cerastes cerastes and Cryptelytrops purpureomaculatus) from various habitats. *Comparative Biochemistry and Physiology Part C: Toxicology & Pharmacology*. 2019; doi: 10.1016/j.cbpc.2019.02.013.

60. Wüster W, Golay P, Warrell DA. Synopsis of recent developments in venomous snake systematics. *Toxicon*. 1997; doi: 10.1016/S0041-0101(96)00152-3.

61. Spawls S, Branch B. The dangerous snakes of Africa: natural history, species directory, venoms, and snakebite. Sanibel Island, FL: Ralph Curtis-Books;

62. Wagstaff SC, Sanz L, Juárez P, Harrison RA, Calvete JJ. Combined snake venomics and venom gland transcriptomic analysis of the ocellated carpet viper, Echis ocellatus. *Journal of Proteomics*. 2009; doi: 10.1016/j.jprot.2008.10.003.

63. Casewell NR, Harrison RA, Wüster W, Wagstaff SC. Comparative venom gland transcriptome surveys of the saw-scaled vipers (Viperidae: Echis) reveal substantial intra-family gene diversity and novel venom transcripts. *BMC Genomics*. 2009; doi: 10.1186/1471-2164-10-564.

64. Arnold N, Robinson M, Carranza S. A preliminary analysis of phylogenetic relationships and biogeography of the dangerously venomous Carpet Vipers, Echis (Squamata, Serpentes, Viperidae) based on mitochondrial DNA sequences. *Amphib Reptilia*. 2009; doi: 10.1163/156853809788201090.

65. Casewell NR, Wagstaff SC, Wüster W, Cook DAN, Bolton FMS, King SI, et al.. Medically important differences in snake venom composition are dictated by distinct postgenomic mechanisms. *Proc Natl Acad Sci USA*. 2014; doi: 10.1073/pnas.1405484111.

66. Barlow A, Pook CE, Harrison RA, Wüster W. Coevolution of diet and prey-specific venom activity supports the role of selection in snake venom evolution. *Proc R Soc B*. 2009; doi: 10.1098/rspb.2009.0048.

67. Richards DP, Barlow A, Wüster W. Venom lethality and diet: Differential responses of natural prey and model organisms to the venom of the saw-scaled vipers (Echis). *Toxicon*. 2012; doi: 10.1016/j.toxicon.2011.10.015.

68. Scott DL, White SP, Otwinowski Z, Yuan W, Gelb MH, Sigler PB. Interfacial Catalysis: The Mechanism of Phospholipase A2. :152012;

69. Rouault M, Rash LD, Escoubas P, Boilard E, Bollinger J, Lomonte B, et al.. Neurotoxicity and Other Pharmacological Activities of the Snake Venom Phospholipase A_2 OS_2: The N-Terminal Region Is More Important Than Enzymatic Activity. *Biochemistry*. 2006; doi: 10.1021/bi060217r.

70. Maraganore JM, Heinrikson RL. The lysine-49 phospholipase A2 from the venom of Agkistrodon piscivorus piscivorus. Relation of structure and function to other phospholipases A2. *Journal of Biological Chemistry*. 1986; doi: 10.1016/S0021-9258(19)89175-5.

71. Wei J-F, Wei X, Chen Q-Y, Huang T, Qiao L-Y, Wang W-Y, et al.. N49 phospholipase A2, a unique subgroup of snake venom group II phospholipase A2. *Biochimica et Biophysica Acta (BBA) - General Subjects*. 2006; doi: 10.1016/j.bbagen.2005.11.022.

72. Manjunatha Kini R. Excitement ahead: structure, function and mechanism of snake venom phospholipase A2 enzymes. *Toxicon*. 2003; doi: 10.1016/j.toxicon.2003.11.002.

73. Lauridsen LP, Laustsen AH, Lomonte B, Gutiérrez JM. Toxicovenomics and antivenom profiling of the Eastern green mamba snake (Dendroaspis angusticeps). *Journal of Proteomics*. 2016; doi: 10.1016/j.jprot.2016.02.003.

74. Conlon JM, Attoub S, Arafat H, Mechkarska M, Casewell NR, Harrison RA, et al.. Cytotoxic activities of [Ser49]phospholipase A2 from the venom of the saw-scaled vipers Echis ocellatus, Echis pyramidum leakeyi, Echis carinatus sochureki, and Echis coloratus. *Toxicon*. 2013; doi: 10.1016/j.toxicon.2013.05.017.

75. Kang TS, Georgieva D, Genov N, Murakami MT, Sinha M, Kumar RP, et al.. Enzymatic toxins from snake venom: structural characterization and mechanism of catalysis: Enzymatic toxins from snake venom. *FEBS Journal*. 2011; doi: 10.1111/j.1742-4658.2011.08115.x.

76. Lee C-Y. Snake Venoms. Berlin/Heidelberg: Springer Berlin Heidelberg;

77. Marsh N, Williams V. Practical applications of snake venom toxins in haemostasis. *Toxicon*. 2005; doi: 10.1016/j.toxicon.2005.02.016.

78. Nielsen VG, Wagner MT, Frank N. Mechanisms Responsible for the Anticoagulant Properties of Neurotoxic Dendroaspis Venoms: A Viscoelastic Analysis. *IJMS*. 2020; doi: 10.3390/ijms21062082.

79. Gasanov SE. Snake Venom Cytotoxins, Phospholipase A2s, and Zn2+-dependent Metalloproteinases: Mechanisms of Action and Pharmacological Relevance. *J Clinic Toxicol*. 2014; doi: 10.4172/2161-0495.1000181.

80. Xiao H, Pan H, Liao K, Yang M, Huang C. Snake Venom PLA_2, a Promising Target for Broad-Spectrum Antivenom Drug Development. *BioMed Research International*. 2017; doi: 10.1155/2017/6592820.

81. Kini RM, Rao VS, Joseph JS. Procoagulant Proteins from Snake Venoms. *Pathophysiol Haemos Thromb*. 2001; doi: 10.1159/000048066.

82. Kini RM. Anticoagulant proteins from snake venoms: structure, function and mechanism. *Biochemical Journal*. 2006; doi: 10.1042/BJ20060302.

83. Labib RS, Azab MH, Farag NW. Effects of Cerastes cerastes (Egyptian sand viper) and Cerastes vipera (Sahara sand viper) snake venoms on blood coagulation: Separation of coagulant and anticoagulant factors and their correlation with arginine esterase and protease activities. *Toxicon*. 1981; doi: 10.1016/0041-0101(81)90120-3.

84. Rogalski A, Soerensen C, op den Brouw B, Lister C, Dashevsky D, Arbuckle K, et al.. Differential procoagulant effects of saw-scaled viper (Serpentes: Viperidae: Echis) snake venoms on human plasma and the narrow taxonomic ranges of antivenom efficacies. *Toxicology Letters*. 2017; doi: 10.1016/j.toxlet.2017.08.020.

85. Kornalik F, Blombäck B. Prothrombin activation induced by ecarin - A prothrombin converting enzyme from echis carinatus venom. *Thrombosis Research*. 1975; doi: 10.1016/0049-3848(75)90150-4.

86. Yamada D, Sekiya F, Morita T. Isolation and Characterization of Carinactivase, a Novel Prothrombin Activator in Echis carinatus Venom with a Unique Catalytic Mechanism. *Journal of Biological Chemistry*. 1996; doi: 10.1074/jbc.271.9.5200.

87. Yamada D, Morita T. Purification and Characterization of a Ca2+-Dependent Prothrombin Activator, Multactivase, from the Venom of Echis multisquamatus. *Journal of Biochemistry*. 1997; doi: 10.1093/oxfordjournals.jbchem.a021862.

88. Kini RM. Structure–function relationships and mechanism of anticoagulant phospholipase A2 enzymes from snake venoms. *Toxicon*. 2005; doi: 10.1016/j.toxicon.2005.02.018.

89. Youngman NJ, Walker A, Naude A, Coster K, Sundman E, Fry BG. Varespladib (LY315920) neutralises phospholipase A2 mediated prothrombinase-inhibition induced by Bitis snake venoms. *Comparative Biochemistry and Physiology Part C: Toxicology & Pharmacology*. 2020; doi: 10.1016/j.cbpc.2020.108818.

Click here to access/download;Table;Table2.pdf

Family	Genus (sub-genus)	Snake	Cell viability assay IC_{50} (µg/mL)	Enzymatic activity assays EC_{50} (µg/mL) PLA$_2$	SVSP	SVMP
Elapidae	*Dendroaspis*	*D. angusticeps*	ND	ND	-	ND
		D. jamesoni	ND	ND	-	241.3 ± 31.1
		D. polylepis	ND	ND	-	ND
		D. viridis	ND	ND	-	135.9 ± 32.2
	Hemachatus	*H. haemachatus*	27.4 ± 2.0	93.7 ± 0.3	-	ND
	Naja (Afronaja)	*N. ashei*	12.1 ± 0.1	38.0 ± 0.1	-	666.6 ± 223.5
		N. katiensis	21.3 ± 0.7	37.1 ± 0.1	-	949.2 ± 395.2
		N. mossambica	18.7 ± 1.3	25.4 ± 0.1	-	428.3 ± 79.7
		N. nigricincta	7.2 ± 0.1	15.5 ± 0.4	-	ND
		N. nigricollis	20.8 ± 1.1	33.5 ± 0.1	-	ND
		N. nubiae	13.6 ± 0.3	20.3 ± 0.1	-	857.5 ± 232.1
		N. pallida	17.5 ± 0.7	17.7 ± 0.5	-	ND
	Naja (Boulengerina)	*N. melanoleuca*	6.9 ± 0.1	80.7 ± 0.4	-	421.3 ± 68.4
	Naja (Uraeus)	*N. anchietae*	15.7 ± 1.4	301.7 ± 0.6	-	ND
		N. annulifera	21.1 ± 1.5	354.8 ± 0.5	-	76.9 ± 9.5
		N. haje	9.9 ± 0.4	249.7 ± 0.6	-	769.6 ± 231.0
		N. nivea	14.5 ± 0.2	705.4 ± 38.8	-	375.6 ± 146.4
		N. senegalensis	4.7 ± 0.2	366.6 ± 10.1	-	ND
Viperidae	*Bitis*	*B. arietans*	5.7 ± 0.3	ND	523.9 ± 45.7	3.3 ± 0.7
		B. gabonica	3.7 ± 0.2	37.0 ± 1.0	104.2 ± 18.0	38.1 ± 7.0
		B. nasicornis	5.5 ± 0.2	35.0 ± 1.0	161.5 ± 20.7	53.9 ± 7.8
		B. rhinoceros	2.8 ± 0.1	ND	1150.0 ± 242.8	69.2 ± 11.7
	Cerastes	*C. cerastes*	2.0 ± 0.1	144.0 ± 7.0	24.8 ± 3.7	57.6 ± 7.1
	Echis	*E. leucogaster*	3.9 ± 0.2	357.0 ± 17.0	ND	13.5 ± 3.7
		E. ocellatus	2.1 ± 0.1	857.0 ± 90.0	ND	2.1 ± 0.4
		E. pyramidum	6.5 ± 0.1	435.0 ± 13.0	ND	9.2 ± 1.2

High activity

Low activity

Not determined (ND)

Not tested

Click here to access/download;Figure;Figure1.pdf

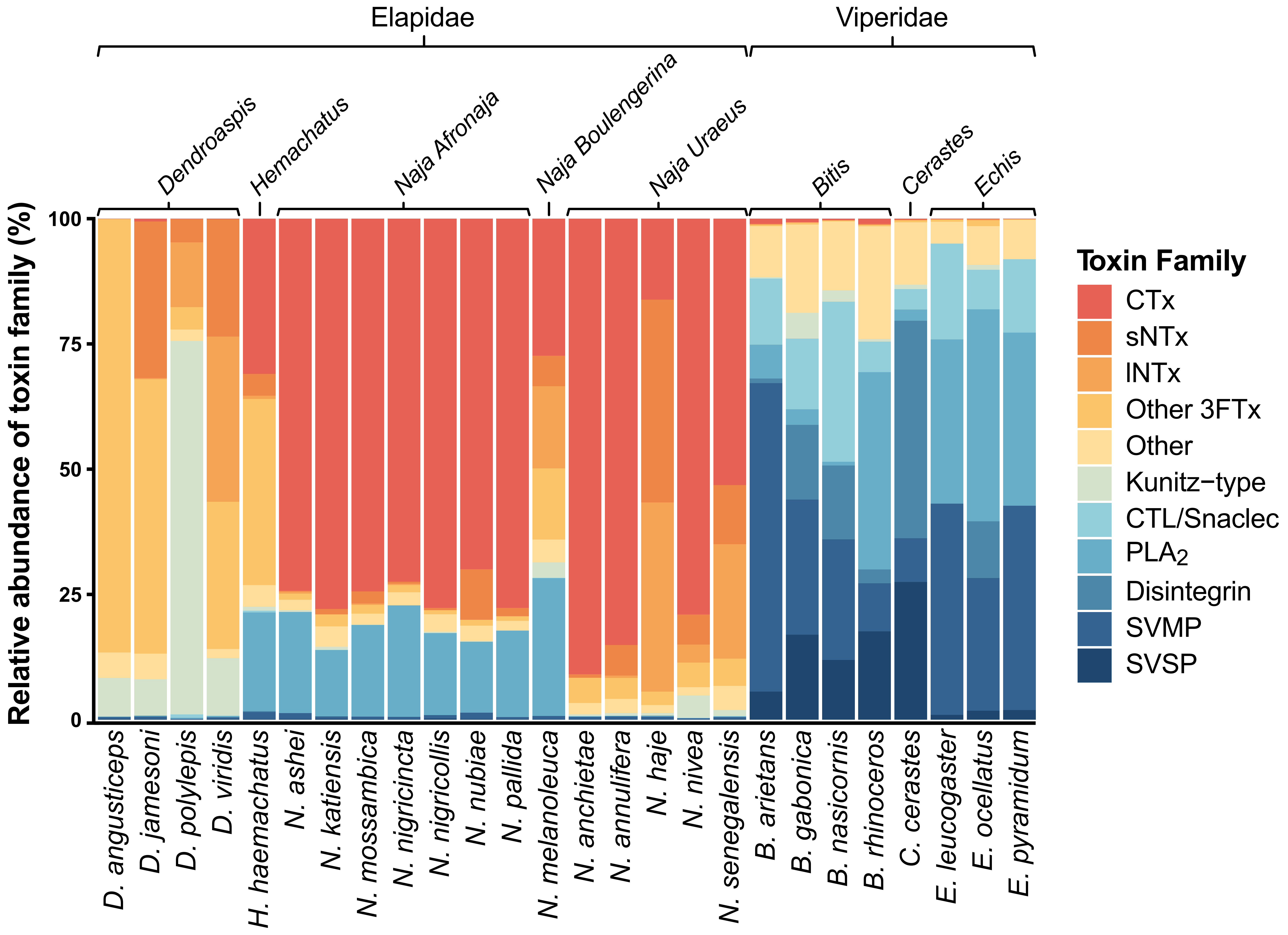

Click here to access/download;Figure;Figure2.pdf

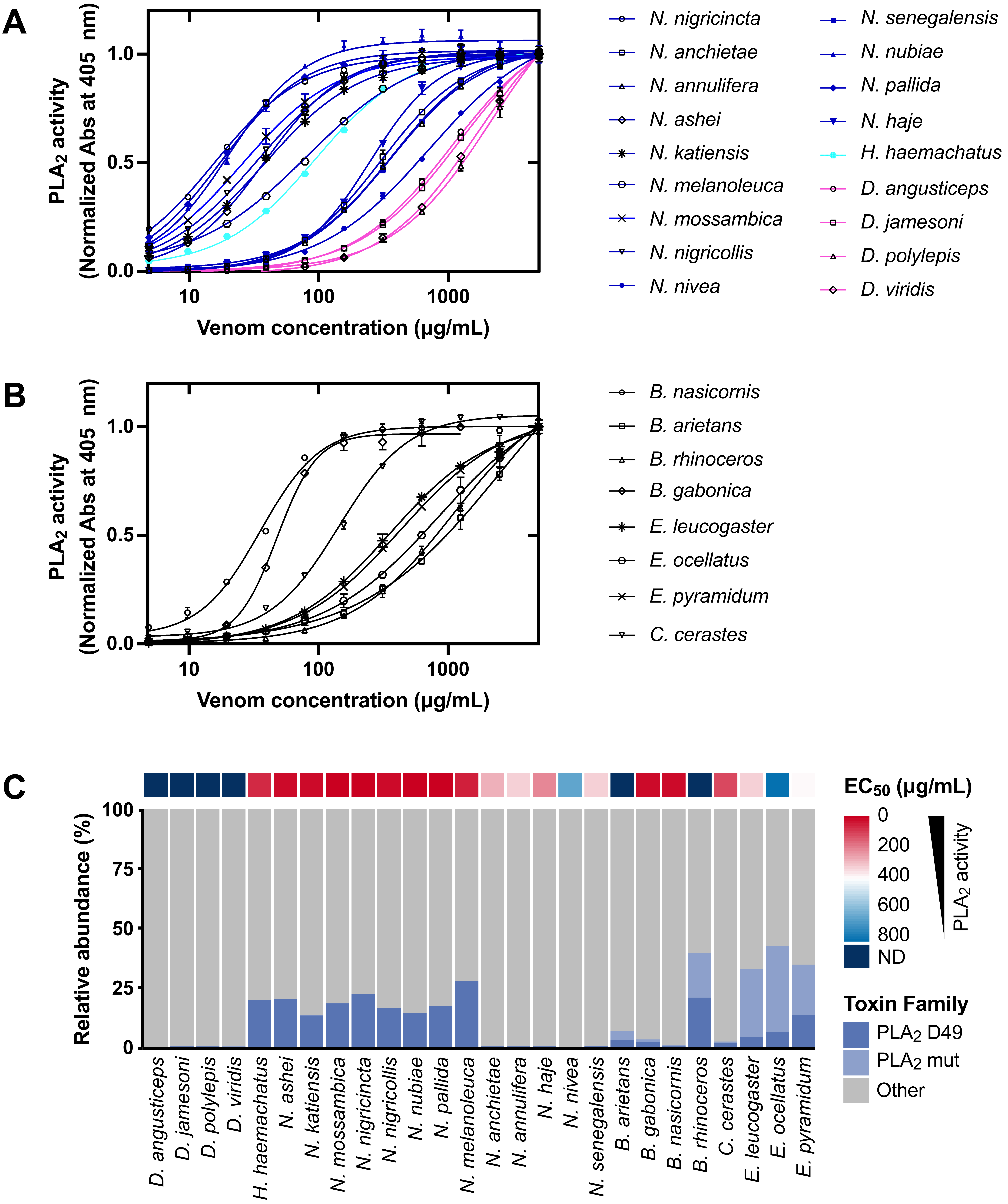

Click here to access/download;Figure;Figure3.pdf

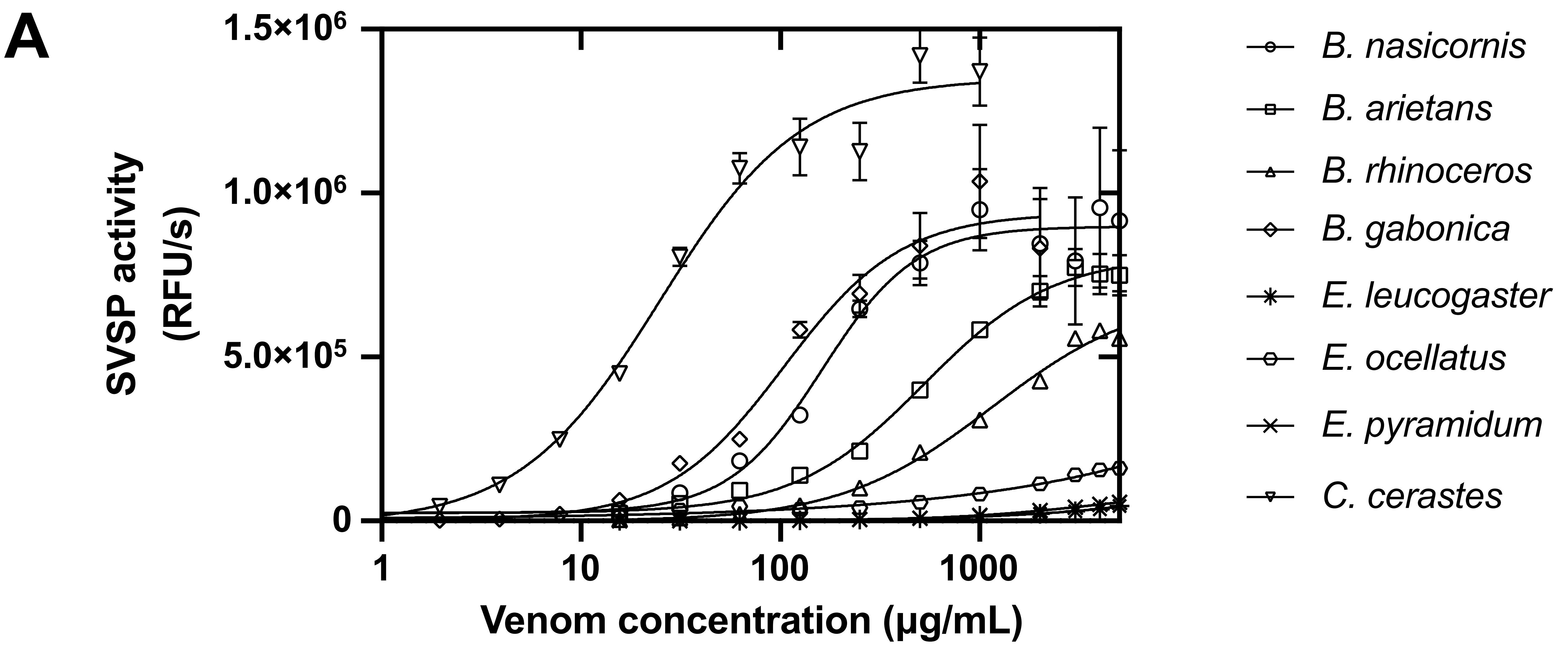

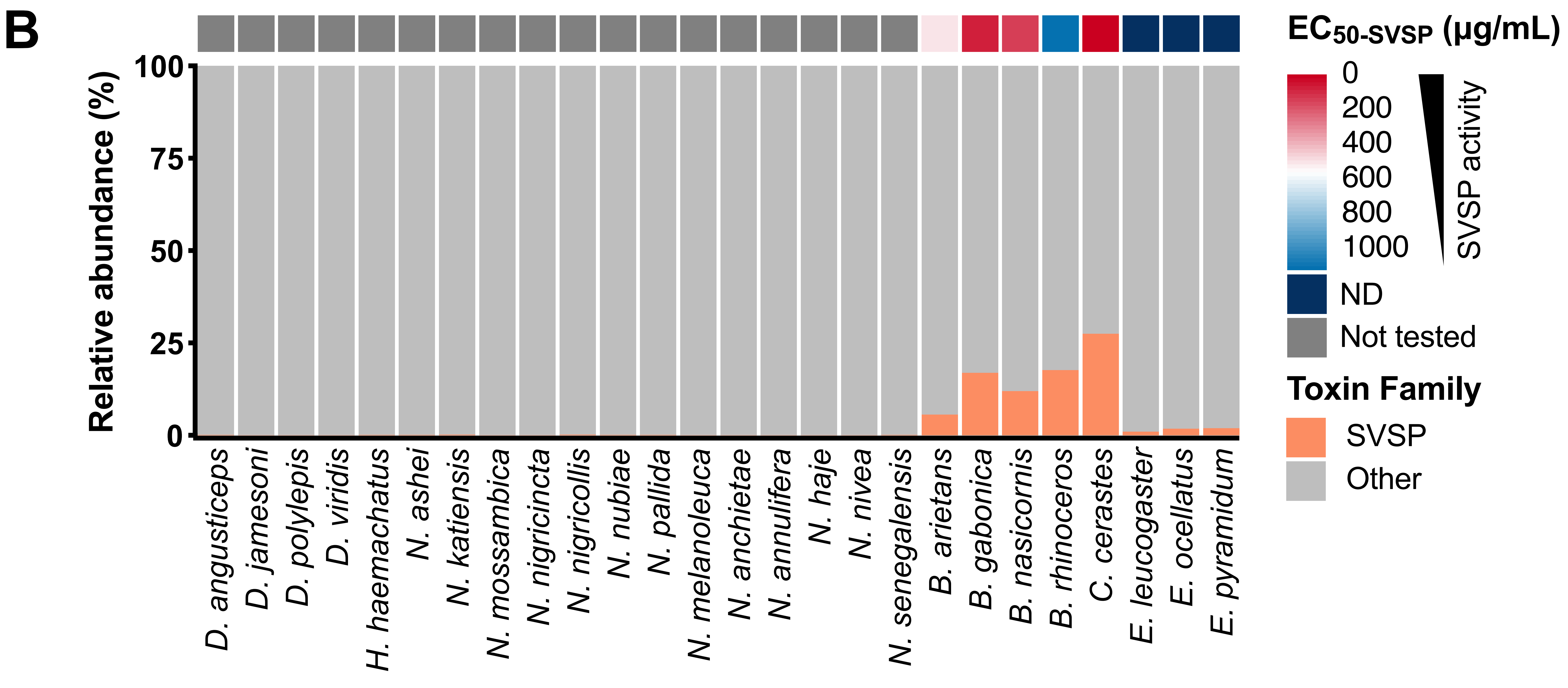

Click here to access/download;Figure;Figure4.pdf

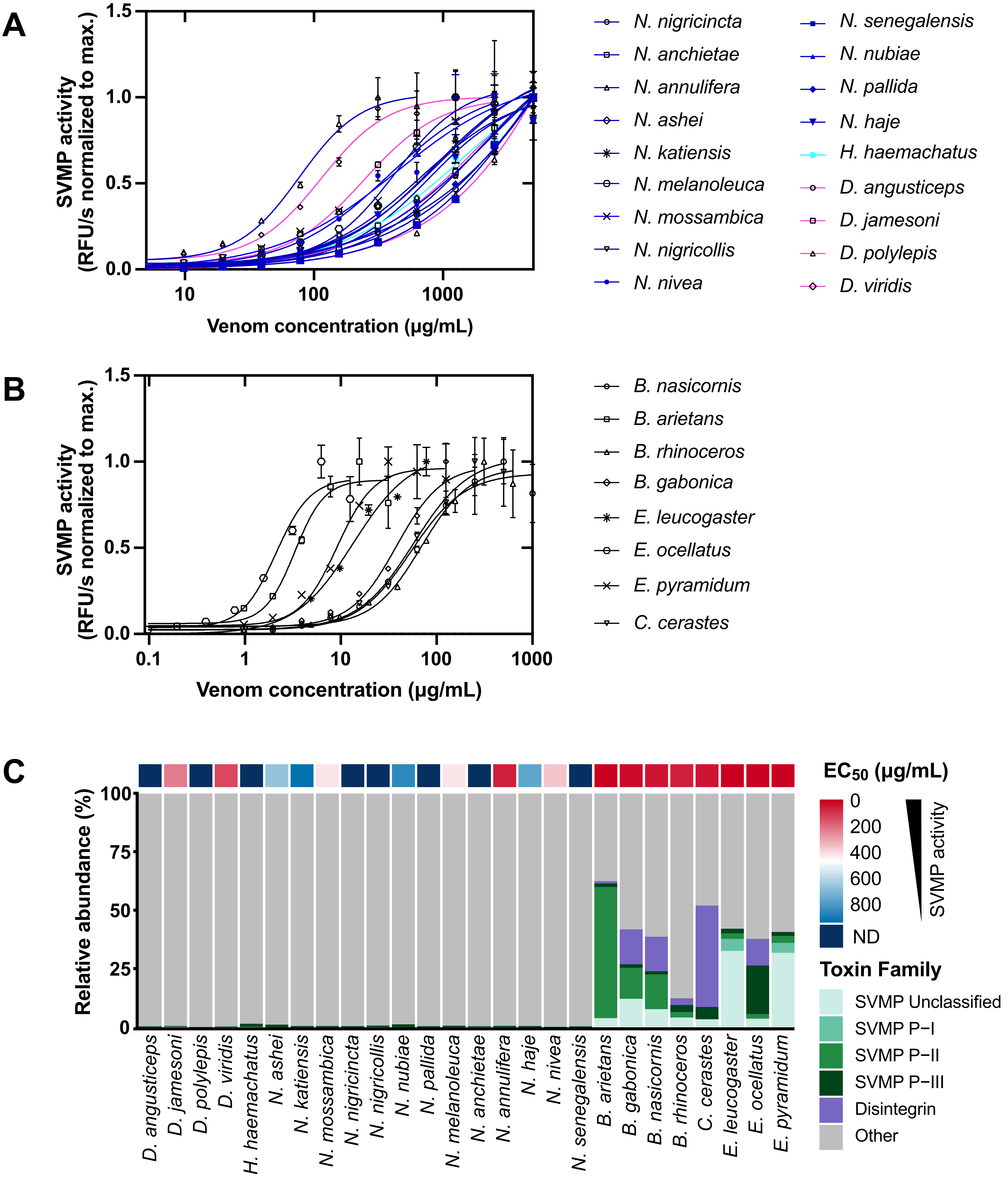

Click here to access/download;Figure;Figure5.pdf

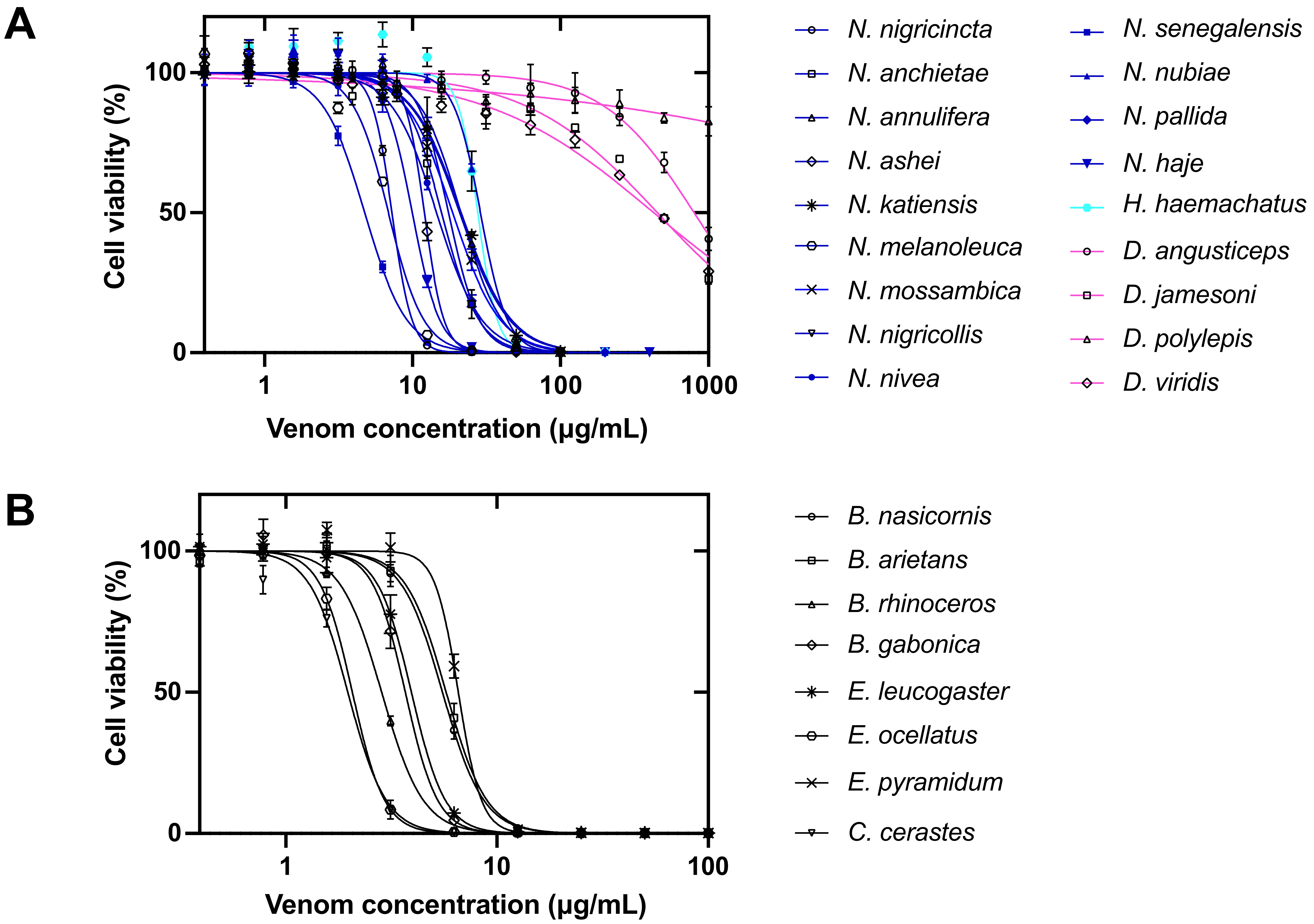

Click here to access/download;Figure;Figure6.pdf

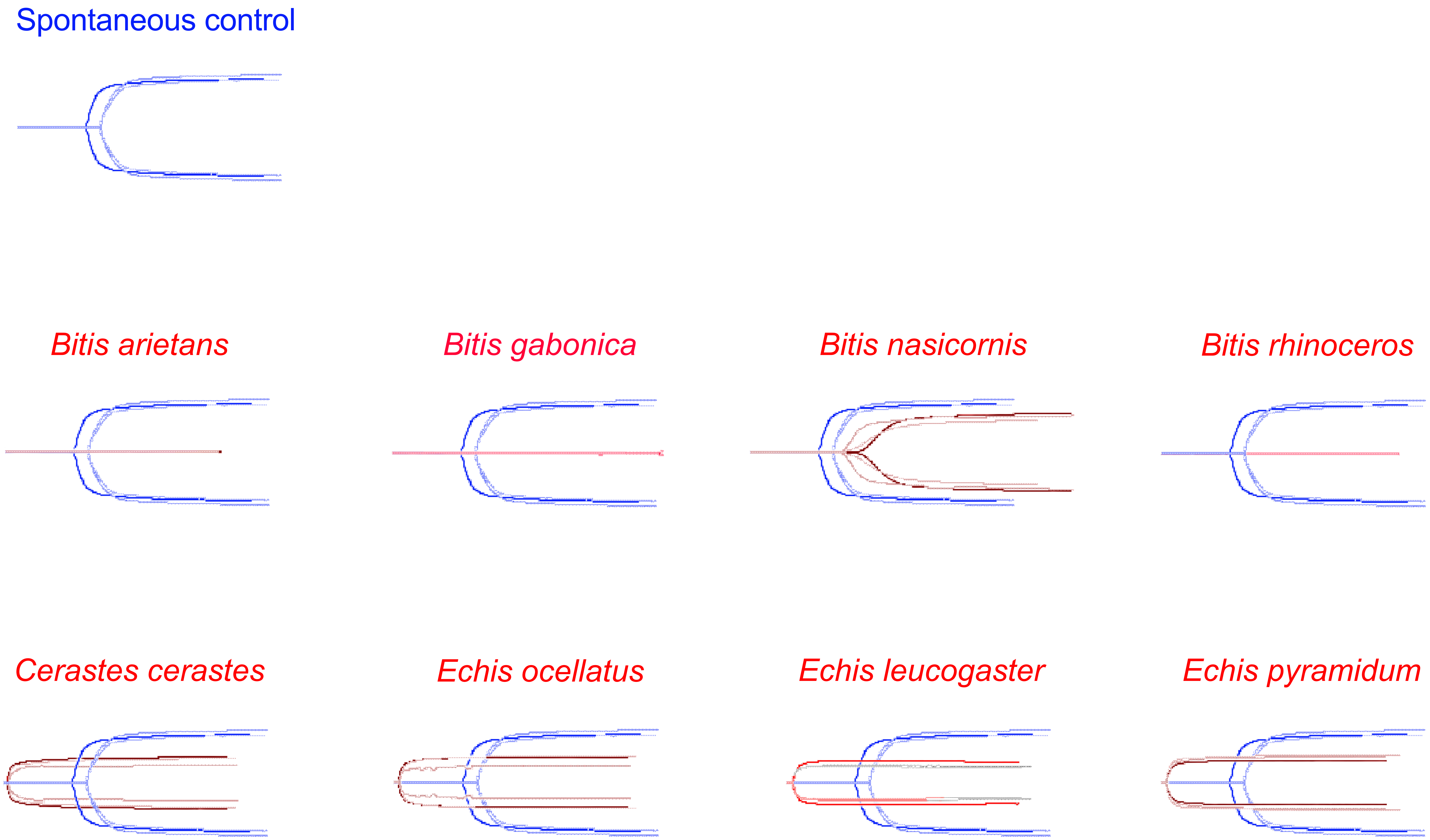

Click here to access/download
Supplementary Material
FigureS1.pdf

Click here to access/download
Supplementary Material
TableS1.xls
