# Supplementary material for: High-throughput proteomics and in vitro functional characterization of the 26 medically most important elapids and vipers from sub-Saharan Africa
Source: Gigascience. 2022 Dec 13;11:giac121. doi: 10.1093/gigascience/giac121 (PMC9744630; doi:10.1093/gigascience/giac121)
Supplement: giac121_Supplemental_Figure_and_Table [file giac121_supplemental_figure_and_table.zip › FigureS1.pdf]

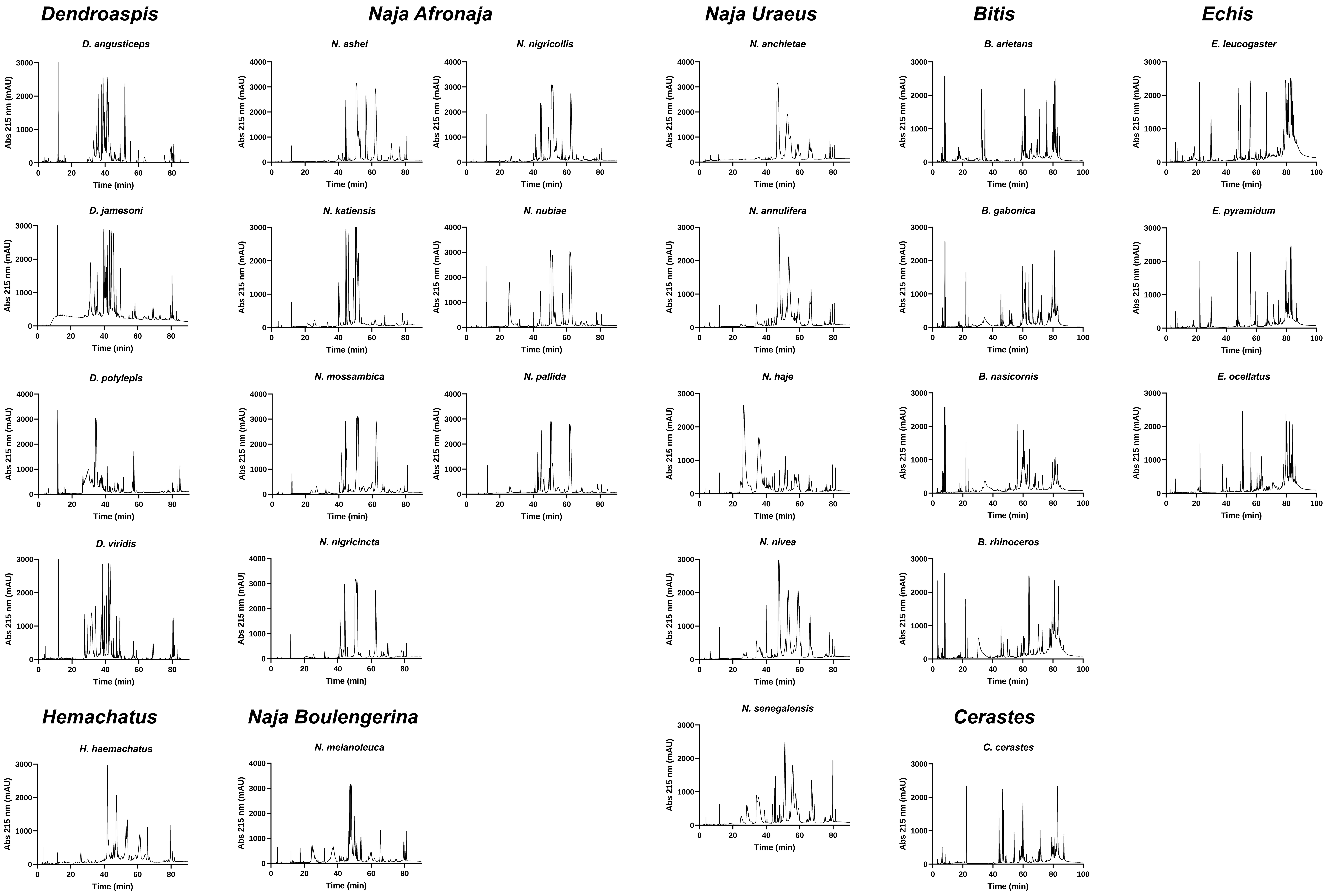
Dendroaspis
D. angusticeps
D. jamesoni
D. polylepis
D. viridis
Naja Afronaja
N. ashei
N. nigricollis
N. katiensis
N. nubiae
N. mossambica
N. pallida
N. nigricincta
Naja Uraeus
N. anchietae
N. annulifera
N. haje
N. nivea
N. senegalensis
Bitis
B. arietans
B. gabonica
B. nasicornis
B. rhinoceros
Echis
E. leucogaster
E. pyramidum
E. ocellatus
Hemachatus
H. haemachatus
Naja Boulengerina
N. melanoleuca
Cerastes
C. cerastes
Abs 215 nm (mAU)
Time (min)
