# Supplementary material for: High-throughput proteomics and in vitro functional characterization of the 26 medically most important elapids and vipers from sub-Saharan Africa
Source: Gigascience. 2022 Dec 13;11:giac121. doi: 10.1093/gigascience/giac121 (PMC9744630; doi:10.1093/gigascience/giac121)
Supplement: giac121_Response_to_Reviewer_Comments_Original_Submission [file giac121_response_to_reviewer_comments_original_submission.pdf]

Author's Response To Reviewer Comments

Close

REVIEWER COMMENTS

Reviewer #1: In this manuscript, the authors present an impressive proteomic and functional dataset of 26 medically significant snake venoms from sub-Saharan Africa. The research is presented from both a medical and evolutionary biology perspective, with the primary methods revolving around RP-HPLC + LC-MS/MS, in vitro enzymatic assays, and cell viability tests performed on venom. The methods are thorough, robust, and repeatable. Altogether, this study presents a concrete foundation for interpreting venom proteomics across the region in a highly consistent manner. Furthermore, this study spans a diverse sampling of taxa which helps unify the previously fragmented proteomic characterizations reported in past studies.

One major area that needs clarification relates to discrepancies in the SVMP contributions reported in this study compared to numerous previous studies, which I have commented on in more detail below. Otherwise, I really enjoyed this paper and would like to extend my compliments to the authors for their robust proteomic methods and clear reporting.

######################

Major comments:

Lines 96 - 97:
Can the authors add some details about how the venom was initially collected, stored, and re-constituted? I assume it was dehydrated and stored at -80 C after collection from the animal, then reconstituted with ultra pure H2O for proteomics and PBS for enzymatics? Were the source animals kept in a facility (i.e., Latoxan) with recognized animal use protocol clearance (IACUC) or similar accredited status? Were venoms representative of individual animals or pooled from several? Was the same source venom aliquot used across treatments per species (i.e., same pool or venom sample)?

Response:
We purchased all whole venoms (pooled from several snakes) in lyophilized form with > 99% purity from Latoxan. Venoms were produced under strictly controlled conditions (https://www.latoxan.com/about_us.php) and Table 1 summarizes information about the origin of the venoms. Venoms were stored at -20 °C and dissolved in assay buffers immediately before use in the experiments, and the same venom vial was used for all assays. Information to clarify this has been added in line 98-100.

Lines 256, 277, and 295:
As the authors pointed out, past studies consistently reported higher contributions of SVMPs in venoms across their sampling. My immediate interpretation coincides with a comment from their conclusion: that this may be an artifact resulting from their usage of the Uniprot database, where lack of close peptide matches to their specific sampling resulted in lower detection rates. This could have influenced the findings otherwise reported by the authors as evolutionary signals or clade specific.
Can the authors add a supplemental table listing reference snake SVMPs which were present in their reference database for identifying matches with Proteome Discoverer (species + sequence IDs or accessions), and point out examples of SVMPs from closely matched species that were present (or lacking)? Are there other well-maintained protein databases (e.g., Genbank) with more closely matched SVMPs that the authors can search against as a test to check if their lower SVMP detection was a reference-based artifact? If this is the case it may be worth also revisiting the other toxin families with a more updated reference database. This is especially true for SVSPs, since their absence in Elapids

influenced the authors decision to leave them out of the enzymatic tests.
Generally speaking, increasing reference database transparency will make this study more accessible to future research attempting to build on their results.

Response:

In the reference UniProt database, SVMPs were available for Echis pyramidum leakeyi, Echis ocellatus, Bitis gabonica, Bitis arietans, Echis pyramidum and Naja mossambica (Table 1). But only three out of 26 snakes had comprehensive coverage of SVMPs where transcriptomic data was included in the database; Bitis gabonica, Echis ocellatus, and Echis pyramidum leakeyi (a sub-species of Echis pyramidum). The low numbers of SVMPs available for these 26 snakes, indicates a need for further work to characterize SVMPs.

With regards to alternative databases, the UniProt database used in this study included unreviewed proteins from the TrEMBL database, which includes, among other databases, translations of coding sequences from NCBI's GenBank. In turn, NCBI's protein database includes sequences from UniProt. In theory, therefore, there should not be much difference between using the NCBI and UniProt databases. However, to test this, we compiled a list of all the SVMPs from the 26 snakes available in each of the databases and compared them. The results are very similar, as shown below, and suggests that the issue with SVMPs is not a UniProt-specific reference artifact, but rather the result of a wider problem with lack of characterization of SVMPs.

We have expanded the conclusion section to mention the low amounts of SVMPs present in both databases. Lines 536-545.

	NCBI	UniProt
Echis pyramidum leakeyi	64	62
Echis ocellatus	46	48
Bitis gabonica	3	3
Bitis arietans	1	2
Echis pyramidum	0	2
Naja mossambica	1	1

Table 1: Number of metalloproteinases available in the NCBI and UniProt protein databases, respectively. If a snake is not mentioned in the table, no metalloproteinase is available.

Lines 256:
Continuing on SVMPs, the authors could add an additional comment in their discussion about SVMPs being one of the larger, more complex venom components ranging from 20-100 kDa (https://doi.org/10.1016/j.toxcx.2020.100052), which makes them especially prone to the effects of otherwise minor inconsistencies between studies.

Response:
We agree with the reviewer and a sentence describing this has been added in line 342-344.

Figure 1: The authors report Disintegrins separately in this figure, however in their conclusion and SVMP enzymatic figure they recognize that Disintegrins overlap with SVMP activity. Does figure 1 lump SVMP Disintegrins in with the SVMPs group, where the lighter blue group labeled "Disintegrins" only includes non-SVMP peptides? Please add a clarification here or group Disintegrins and SVMPs together and report them as combined.

Response:
In both Figure 1 and Figure 4 (and throughout the paper) SVMP disintegrins are included in the SVMP group. The disintegrin group contains only non-SVMP disintegrins which is in line with UniProt classifications. We have added two sentences to the legends of Figure 1 and Figure 4 to clarify this.

Minor comments:

Line 82: add "high-throughput" to venomic studies.

Response:
Regardless if previous studies have been run in high or low throughput they have mainly focused on one or a few species. Therefore, the word "high-throughput" is not added.

Line 117: If known, can the authors please add what temperature the venom fractions were dried at using the vacuum centrifuge.

Response:
The sentence of collecting the fractions is removed, since the fractions were not used for any work in this study.

Line 149: If known, can the authors please add the date (month) which the 331,759 sequences were downloaded or otherwise utilized from Uniprot?

Response:
The date (May 2022), has been added in line 152.

Line 175: Please change "set up" to "run" or "performed".

Response:
Changed to “performed”

Line 350: Can the authors clarify here that some SVMPs are disintegrin containing, and how this may affect this discrepancy? (https://doi.org/10.1016/j.toxcx.2020.100052). I recognize that the authors mention it briefly in their conclusion but I believe it is also important in this context as well.

Response:
We thank the reviewer for pointing this out and agree, one potential explanation for this discrepancy, could have been that the identified peptides may have been classified incorrectly as disintegrins instead of as SVMPs. However, we looked into this further and found that this is unlikely to be the case here. As no SVMP for C. cerastes exists in UniProt, we cannot rule this out completely but the disintegrins we identified are the same disintegrins identified in previous studies, and the peptides map exactly to disintegrins. We have added a sentence to line 361-365 to clarify this.

Reviewer #2: In this work, Nguyen et al. describe a comprehensive proteomics and functional characterization of the 26 most medically important venomous snake species in Sub-Saharan Africa. Therefore, Nguyen and coworkers performed in-solution digestion combined with subsequent mass spectrometric identification of toxin peptides. In vitro functional assays were performed to characterize the toxicity of different toxin families (eg. svMP, svSP) in crude venom. The manuscript is generally well-written and easy to follow, and the data are presented clearly and logically. The experiments appear to have been done with great care, and the data analysis is rigorous. I have only some minor comments listed below to avoid any inconsistencies.

Line 41 f: The authors stated that venomous snakes inflict approximately 500,000 bites in Africa. The respective literature is from 2008 and I was wondering if there weren't more recent case numbers and literature.

Response:
According to WHO in 2021, in Africa there are an estimated 435 000 to 580 000 snake bites annually that need treatment (https://www.who.int/news-room/fact-sheets/detail/snakebite-envenoming#:~:text=In%20Asia%20up%20to%202,bites%20annually%20that%20need%20treatment.) We have also added a more recent reference to the paper (Gutiérrez, J. et el. Nat Rev Dis Primers, 2017.

Line 103 ff (Table 1): In the introduction, authors discussed the difficulty of data harmonization due to different laboratories using multiple protocols. I absolutely agree with that statement but please be aware also of the origin and composition of the respective venom samples. Here, samples were purchased by a

company without any further details instead of sampling in respective distribution areas in Sub-Saharan Africa. This should in no way detract this work, but authors should be are of that venom composition can vary to samples of venomous snakes in their natural habitat. The authors should emphasize this clearly and discuss it in the context of differences in venom composition also to previous studies.

Response:
We thank the reviewer for this comment and totally agree. We discuss this in lines 261-263, 340-341, 377-378.

Line 108 ff: I was wondering why the authors mentioned the venom fractionation in the material and method section. The authors used for proteomics and in vitro characterization whole venom samples instead of pre-fractionated samples. I would be happy when the authors could comment on this.

Response:
We provided RP-HPLC chromatograms of the 26 snake venoms in the Supplementary Figure S1. This is now stated in line 113, and the sentence referring to fractions has been removed.

Line 227 f: The authors stated: "…calculation of protein family abundances as a percentage of total identified proteins (mol/mol)" - Did the authors really mean (mol/mol)? If yes, can you please briefly explain it.

Response:
The method of quantification used here, label free quantification on the mass spectrometer, provides results in mol/mol. The composition is represented in terms of moles, which takes into account the size (molecular weight) of the protein. With other quantification methods in venomics, often, w/w is used which gives the composition in terms of mass. Therefore, we think it is important to specify that mol/mol is used here.

Line 272 & 275: The authors showed that the venom of Naja haje contained mainly sNTxs (40%) and lNTxs (38%) and stated to confirm previous studies. However, from previous and our own investigations to this species, we know that the CTx content is in fact much higher than the authors stated. I would ask the authors to check again the literature for all analyzed species in detail to be sure for any differences and similarities.

Response:
We thank the reviewer for pointing this out, it has been corrected in line 275-276.

Figure 2: The color code used for the different toxin families is not optimal for printouts. The subtle hue gradations between families are difficult to distinguish. This is not a major problem when viewed online, as it can be zoomed in and the contrast for the gradations is much better, but I would recommend the authors redistribute the colors so that the difference can be seen on printouts as well.

Response:
The colors of the bar chart in Figure 2C have been changed to increase the contrast between the two groups.

Close
