# Supplementary material for: High-throughput proteomics and in vitro functional characterization of the 26 medically most important elapids and vipers from sub-Saharan Africa
Source: Gigascience. 2022 Dec 13;11:giac121. doi: 10.1093/gigascience/giac121 (PMC9744630; doi:10.1093/gigascience/giac121)
Supplement: giac121_Reviewer_1_Report_Original_Submission — Michael Hogan -- 8/30/2022 Reviewed [file giac121_reviewer_1_report_original_submission.pdf]

Reviewer Report

Title: High-throughput proteomics and in vitro functional characterization of the 26 medically most important elapids and vipers from sub-Saharan Africa

Version: Original Submission **Date:** 8/30/2022

Reviewer name: Michael Hogan

Reviewer Comments to Author:

In this manuscript, the authors present an impressive proteomic and functional dataset of 26 medically significant snake venoms from sub-Saharan Africa. The research is presented from both a medical and evolutionary biology perspective, with the primary methods revolving around RP-HPLC + LC-MS/MS, in vitro enzymatic assays, and cell viability tests performed on venom. The methods are thorough, robust, and repeatable. Altogether, this study presents a concrete foundation for interpreting venom proteomics across the region in a highly consistent manner. Furthermore, this study spans a diverse sampling of taxa which helps unify the previously fragmented proteomic characterizations reported in past studies.

One major area that needs clarification relates to discrepancies in the SVMP contributions reported in this study compared to numerous previous studies, which I have commented on in more detail below. Otherwise, I really enjoyed this paper and would like to extend my compliments to the authors for their robust proteomic methods and clear reporting.

######################

Major comments:

Lines 96 - 97:

Can the authors add some details about how the venom was initially collected, stored, and re-constituted? I assume it was dehydrated and stored at -80 C after collection from the animal, then reconstituted with ultra pure H_2O for proteomics and PBS for enzymatics? Were the source animals kept in a facility (i.e., Latoxan) with recognized animal use protocol clearance (IACUC) or similar accredited status? Were venoms representative of individual animals or pooled from several? Was the same source venom aliquot used across treatments per species (i.e., same pool or venom sample)?

Lines 256, 277, and 295:

As the authors pointed out, past studies consistently reported higher contributions of SVMPs in venoms across their sampling. My immediate interpretation coincides with a comment from their conclusion: that this may be an artifact resulting from their usage of the Uniprot database, where lack of close peptide matches to their specific sampling resulted in lower detection rates. This could have influenced the findings otherwise reported by the authors as evolutionary signals or clade specific.

Can the authors add a supplemental table listing reference snake SVMPs which were present in their reference database for identifying matches with Proteome Discoverer (species + sequence IDs or accessions), and point out examples of SVMPs from closely matched species that were present (or lacking)? Are there other well-maintained protein databases (e.g., Genbank) with more closely matched SVMPs that the authors can search against as a test to check if their lower SVMP detection was a

reference-based artifact? If this is the case it may be worth also revisiting the other toxin families with a more updated reference database. This is especially true for SVSPs, since their absence in Elapids influenced the authors decision to leave them out of the enzymatic tests.
Generally speaking, increasing reference database transparency will make this study more accessible to future research attempting to build on their results.
Lines 256:
Continuing on SVMPs, the authors could add an additional comment in their discussion about SVMPs being one of the larger, more complex venom components ranging from 20-100 kDa (https://doi.org/10.1016/j.toxcx.2020.100052), which makes them especially prone to the effects of otherwise minor inconsistencies between studies.
Figure 1: The authors report Disintegrins separately in this figure, however in their conclusion and SVMP enzymatic figure they recognize that Disintegrins overlap with SVMP activity. Does figure 1 lump SVMP Disintegrins in with the SVMPs group, where the lighter blue group labeled "Disintegrins" only includes non-SVMP peptides? Please add a clarification here or group Disintegrins and SVMPs together and report them as combined.
######################
Minor comments:
Line 82: add "high-throughput" to venomic studies.
Line 117: If known, can the authors please add what temperature the venom fractions were dried at using the vacuum centrifuge.
Line 149: If known, can the authors please add the date (month) which the 331,759 sequences were downloaded or otherwise utilized from Uniprot?
Line 175: Please change "set up" to "run" or "performed".
Line 350: Can the authors clarify here that some SVMPs are desintegrin containing, and how this may affect this discrepancy? (https://doi.org/10.1016/j.toxcx.2020.100052). I recognize that the authors mention it briefly in their conclusion but I believe it is also important in this context as well.

Methods

Are the methods appropriate to the aims of the study, are they well described, and are necessary controls included? Choose an item.

Conclusions

Are the conclusions adequately supported by the data shown? Choose an item.

Reporting Standards

Does the manuscript adhere to the journal's guidelines on minimum standards of reporting? Choose an item.

Choose an item.

Statistics

Are you able to assess all statistics in the manuscript, including the appropriateness of statistical tests used? Choose an item.

Quality of Written English

Please indicate the quality of language in the manuscript: Choose an item.

Declaration of Competing Interests

Please complete a declaration of competing interests, considering the following questions:

- Have you in the past five years received reimbursements, fees, funding, or salary from an organisation that may in any way gain or lose financially from the publication of this manuscript, either now or in the future?
- Do you hold any stocks or shares in an organisation that may in any way gain or lose financially from the publication of this manuscript, either now or in the future?
- Do you hold or are you currently applying for any patents relating to the content of the manuscript?
- Have you received reimbursements, fees, funding, or salary from an organization that holds or has applied for patents relating to the content of the manuscript?
- Do you have any other financial competing interests?
- Do you have any non-financial competing interests in relation to this paper?

If you can answer no to all of the above, write 'I declare that I have no competing interests' below. If your reply is yes to any, please give details below.

I declare that I have no competing interests

I agree to the open peer review policy of the journal. I understand that my name will be included on my report to the authors and, if the manuscript is accepted for publication, my named report including any attachments I upload will be posted on the website along with the authors' responses. I agree for my report to be made available under an Open Access Creative Commons CC-BY license (http://creativecommons.org/licenses/by/4.0/). I understand that any comments which I do not wish to be included in my named report can be included as confidential comments to the editors, which will not be published.

Choose an item.

To further support our reviewers, we have joined with Publons, where you can gain additional credit to further highlight your hard work (see: https://publons.com/journal/530/gigascience). On publication of this paper, your review will be automatically added to Publons, you can then choose whether or not to claim your Publons credit. I understand this statement.

Yes Choose an item.
