# Supplementary material for: High-throughput proteomics and in vitro functional characterization of the 26 medically most important elapids and vipers from sub-Saharan Africa
Source: Gigascience. 2022 Dec 13;11:giac121. doi: 10.1093/gigascience/giac121 (PMC9744630; doi:10.1093/gigascience/giac121)
Supplement: giac121_Reviewer_2_Report_Original_Submission — Benjamin-Florian Hempel -- 9/13/2022 Reviewed [file giac121_reviewer_2_report_original_submission.pdf]

Reviewer Report

Title: High-throughput proteomics and in vitro functional characterization of the 26 medically most important elapids and vipers from sub-Saharan Africa

Version: Original Submission **Date:** 9/13/2022

Reviewer name: Benjamin-Florian Hempel

Reviewer Comments to Author:

In this work, Nguyen et al. describe a comprehensive proteomics and functional characterization of the 26 most medically important venomous snake species in Sub-Saharan Africa. Therefore, Nguyen and coworkers performed in-solution digestion combined with subsequent mass spectrometric identification of toxin peptides. In vitro functional assays were preformed to characterize the toxicity of different toxin families (eg. svMP, svSP) in crude venom. The manuscript is generally well-written and easy to follow, and the data are presented clearly and logically. The experiments appear to have been done with great care, and the data analysis is rigorous. I have only some minor comments listed below to avoid any inconsistencies.

Line 41 f: The authors stated that venomous snakes inflict approximately 500,000 bites in Africa. The respective literature is from 2008 and I was wondering if there weren't more recent case numbers and literature.

Line 103 ff (Table 1): In the introduction, authors discussed the difficulty of data harmonization due to different laboratories using multiple protocols. I absolutely agree with that statement but please be aware also of the origin and composition of the respective venom samples. Here, samples were purchased by a company without any further details instead of sampling in respective distribution areas in Sub-Saharan Africa. This should in no way detract this work, but authors should be are of that venom composition can vary to samples of venomous snakes in their natural habitat. The authors should emphasize this clearly and discuss it in the context of differences in venom composition also to previous studies.

Line 108 ff: I was wondering why the authors mentioned the venom fractionation in the material and method section. The authors used for proteomics and in vitro characterization whole venom samples instead of pre-fractionated samples. I would be happy when the authors could comment on this.

Line 227 f: The authors stated: "…calculation of protein family abundances as a percentage of total identified proteins (mol/mol)" - Did the authors really mean (mol/mol)? If yes, can you please briefly explain it.

Line 272 & 275: The authors showed that the venom of Naja haje contained mainly sNTxs (40%) and lNTxs (38%) and stated to confirm previous studies. However, from previous and our own investigations to this species, we know that the CTx content is in fact much higher than the authors stated. I would ask the authors to check again the literature for all analyzed species in detail to be sure for any differences and similarities.

Figure 2: The color code used for the different toxin families is not optimal for printouts. The subtle hue gradations between families are difficult to distinguish. This is not a major problem when viewed online,

as it can be zoomed in and the contrast for the gradations is much better, but I would recommend the authors redistribute the colors so that the difference can be seen on printouts as well.

Methods

Are the methods appropriate to the aims of the study, are they well described, and are necessary controls included? Choose an item.

Conclusions

Are the conclusions adequately supported by the data shown? Choose an item.

Reporting Standards

Does the manuscript adhere to the journal's guidelines on minimum standards of reporting? Choose an item.

Choose an item.

Statistics

Are you able to assess all statistics in the manuscript, including the appropriateness of statistical tests used? Choose an item.

Quality of Written English

Please indicate the quality of language in the manuscript: Choose an item.

Declaration of Competing Interests

Please complete a declaration of competing interests, considering the following questions:

- Have you in the past five years received reimbursements, fees, funding, or salary from an organisation that may in any way gain or lose financially from the publication of this manuscript, either now or in the future?
- Do you hold any stocks or shares in an organisation that may in any way gain or lose financially from the publication of this manuscript, either now or in the future?
- Do you hold or are you currently applying for any patents relating to the content of the manuscript?
- Have you received reimbursements, fees, funding, or salary from an organization that holds or has applied for patents relating to the content of the manuscript?
- Do you have any other financial competing interests?
- Do you have any non-financial competing interests in relation to this paper?

If you can answer no to all of the above, write 'I declare that I have no competing interests' below. If your reply is yes to any, please give details below.

I declare that I have no competing interests

I agree to the open peer review policy of the journal. I understand that my name will be included on my report to the authors and, if the manuscript is accepted for publication, my named report including any attachments I upload will be posted on the website along with the authors' responses. I agree for my report to be made available under an Open Access Creative Commons CC-BY license (http://creativecommons.org/licenses/by/4.0/). I understand that any comments which I do not wish to be included in my named report can be included as confidential comments to the editors, which will not be published.

Choose an item.

To further support our reviewers, we have joined with Publons, where you can gain additional credit to further highlight your hard work (see: https://publons.com/journal/530/gigascience). On publication of this paper, your review will be automatically added to Publons, you can then choose whether or not to claim your Publons credit. I understand this statement.

Yes Choose an item.
